# Bioinformatics based exploration of the anti-NAFLD mechanism of Wang’s empirical formula via TLR4/NF-κB/COX2 pathway

**DOI:** 10.1186/s10020-024-01022-3

**Published:** 2024-12-27

**Authors:** Suhong Chen, Chuanjie Zhou, Jiahui Huang, Yunlong Qiao, Ning Wang, Yuzhen Huang, Bo Li, Wanfeng Xu, Xinglishang He, Kungen Wang, Yihui Zhi, Guiyuan Lv, Shuhua Shen

**Affiliations:** 1 Collaborative Innovation Center of Yangtze River Delta Region Green Pharmaceuticals, Zhejiang University of Technology, No. 18, Chaowang Road, Gongshu District, Hangzhou, 310014 Zhejiang China; 2 Disease Prevention and Health Management Center, The First Affiliated Hospital of Zhejiang Chinese Medical University, Hangzhou, 310006 Zhejiang China; 3 Zhejiang Provincial Key Laboratory of TCM for Innovative R & D and Digital Intelligent Manufacturing of TCM Great Health Products. Huzhou, Zhejiang, 313200 China; 4 College of Pharmaceutical Science, Zhejiang Chinese Medical University, No. 548, Binwen Road, Biniang District, Hangzhou, 310053 Zhejiang China; 5 Kun-Gen Wang National Famous Chinese Medicine Doctor Studio, Hangzhou, 310006 Zhejiang China

**Keywords:** Nonalcoholic fatty liver disease, Wang’s empirical formula, Liver inflammation, HPLC-Q-TOF/MS, Bioinformatics, Network pharmacology

## Abstract

**Background:**

Nonalcoholic fatty liver disease (NAFLD) has developed as a leading public wellness challenge as a result of changes in dietary patterns. Unfortunately, there is still a lack of effective pharmacotherapy methods for NAFLD. Wang’s empirical formula (WSF) has demonstrated considerable clinical efficacy in treating metabolic disorders for years. Nevertheless, the protective effect of WSF against NAFLD and its underlying mechanism remains poorly understood.

**Methods:**

The NAFLD model was established using a 17-week high-sucrose and high-fat (HSHF) diet with 32 ICR mice. In assessing the therapeutic efficacy of WSF on NAFLD, we detected changes in body weight, viscera weight, biomarkers of glycolipid metabolism in serum and liver, transaminase levels and histopathology of liver with H&E and Oil Red O staining after oral administration. The chemical components in WSF were extensively identified and gathered utilizing the HPLC-Q-TOF/MS system, database mining from HMDB, MassBank, and TCMSP databases, alongside literature searches from CNKI, Wanfang and VIP databases. The forecast of network pharmacology approach was then utilized to investigate the probable mechanisms by which WSF improves NAFLD based on the performance of prospective target identification and pathway enrichment analysis. Besides, molecular docking was also conducted for the verification of combination activities between active components of WSF and core proteins related to NAFLD. In final, validation experiments of obtained pathways were conducted through ELISA, immunohistochemistry (IHC), and western blot (WB) analysis.

**Results:**

Pharmacodynamic outcomes indicated that WSF intervention effectively mitigated obesity, fat accumulation in organs, lipid metabolism disorders, abnormal transaminase levels and liver pathology injury in NAFLD mice (*P* < 0.05, 0.01). A total of 72 existent ingredients of WSF were acquired by HPLC-Q-TOF/MS and database, and 254 common targets (11.6% in total targets) of NAFLD and WSF were identified. Network pharmacology revealed that WSF presses NAFLD via modulating TNF, IL6, AKT1, IL1B, PTGS2 (COX2), and other targets, and the probable pathways were primarily inflammatory signaling pathways, as confirmed by molecular docking. Molecular biology experiments further conformed that WSF could decrease levels of inflammatory factors like IL-1β, IL-6 and TNF-α (*P* < 0.01) and expression of TLR4, NF-κB and COX-2 (*P* < 0.05, 0.01) in the liver.

**Conclusion:**

WSF treatment effectively protects against lipid metabolism disorders and liver inflammation injury in HSHF diet-induced NAFLD mice, and its molecular mechanism might be via suppressing the TLR4/NF-κB/COX-2 inflammatory pathway to reduce the release of inflammatory cytokines in the liver.

**Graphical abstract:**

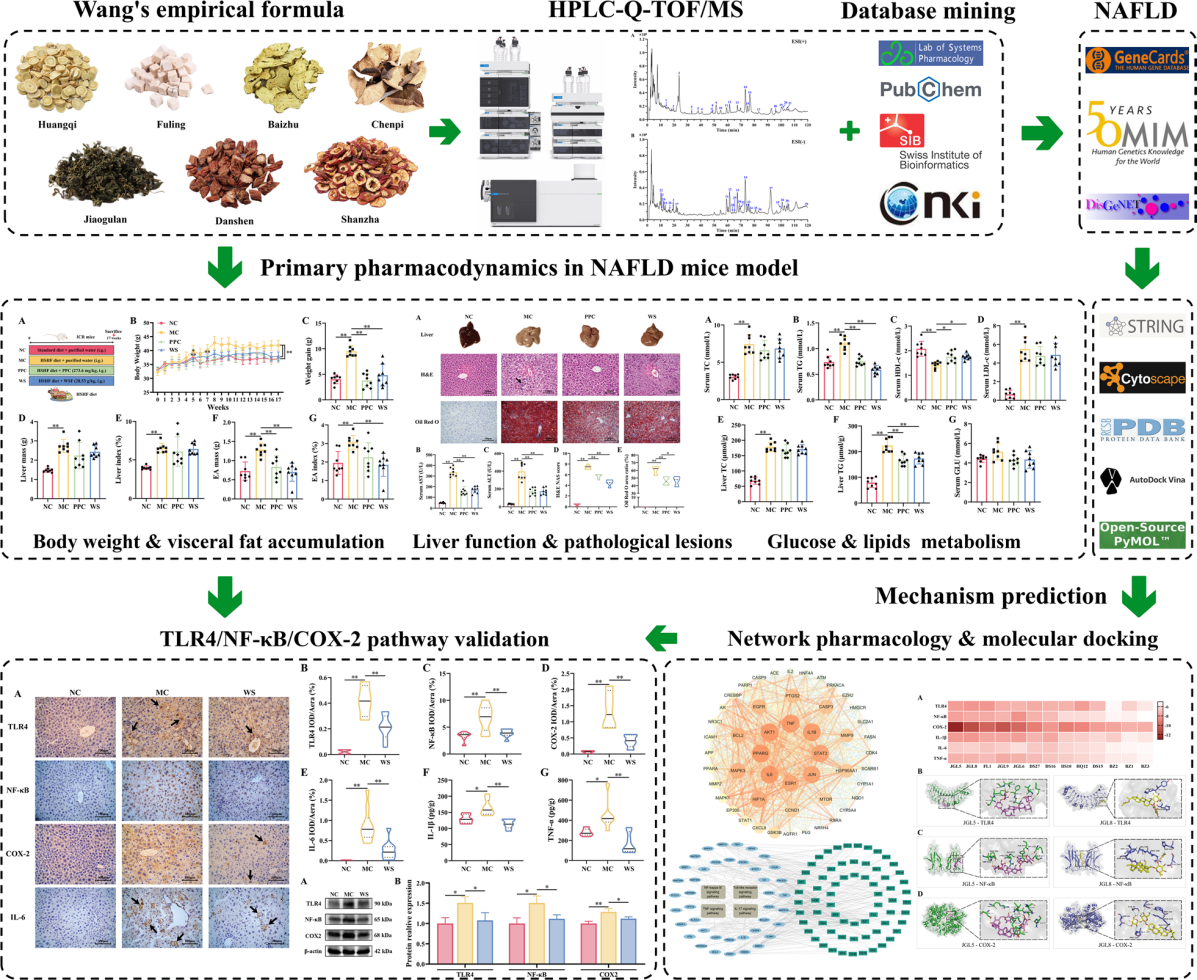

**Supplementary Information:**

The online version contains supplementary material available at 10.1186/s10020-024-01022-3.

## Introduction

Due to alterations in dietary patterns, nonalcoholic fatty liver disease (NAFLD) caused by overnutrition and metabolic disorders has become a common chronic liver disease globally (Younossi et al. 2018). NAFLD is appraised to afflict approximately 25% of the world population with a gradual upward trend (Younossi et al. 2019). The total adult NAFLD prevalence have been reported as high as 29.81% in China, and it is predicated that there will be 314.58 million NAFLD patients in 2030 if without effective control (Li et al. 2019a, b; Zhou et al. 2019). NAFLD is typically divided into two subtypes: nonalcoholic fatty liver (NAFL, with mild symptoms) and nonalcoholic steatohepatitis (NASH, with steatosis of hepatocytes, aggravated inflammatory reaction, and irreversible hepatocyte damage like ballooning transformation) (Loomba et al. 2021). Progression from NAFLD can lead to liver fibrosis, cirrhosis and even liver cancer at a later stage, causing a tremendous burden on human health. Therefore, a penetrative study on the pathophysiology and management of NAFLD has important practical significance.

At present, there is still a lack of effective pharmacotherapy methods for NAFLD (Tilg et al. 2023). Instead, it is recommended to change unhealthy lifestyle like improving dietary structure and lifestyle modifications such as exercise to mitigate the progression of NAFLD firstly (Houttu et al. 2021; Xiong et al. 2021). The mainstay of drug treatments for NAFLD lies in the inhibition of lipid accumulation and protection of liver damage (include hepatic fibrosis). However, some drugs suffer from incomplete therapeutic efficacy or adverse reactions during clinical use, for instance statins might cause elevations of hepatic aminotransferase levels, high risks of new-onset diabetes, myopathy and even myalgias or rhabdomyolysis (Dong et al. 2014). Therefore, search for safer and more effective drugs for NAFLD treatment is imperative.

Traditional Chinese medicine (TCM) is recognized for its unique action modes of multi-target and multi-channel, and there is plenty of testimony that TCM plays a significant role in NAFLD prevention and treatment (Shi et al. 2020; Chen et al. 2021a, b). Wang’s empirical formula (WSF) was a clinical empirical formula developed for the remedy of metabolic diseases by Prof. Wang Kun-Gen, the first national famous doctor of TCM and national academic leader of “spleen and stomach disease of TCM” (Shen et al. 2018). WSF is derived from the classic traditional formula Er-Chen Decoction, and is composed of *Gynostemmae Herba* (Jiaogulan), *Astragali Radix* (Huangqi), *Crataegi Fructus* (Shanzha), *Salviae Miltiorrhizae Radix et Rhizoma* (Danshen), and *Poria* (Fuling), etc. Meanwhile, WSF has the impact of “invigorating the spleen and benefiting the stomach, clearing heat and promoting diuresis”, which is congruent with the TCM’s therapy theory for NAFLD. Despite WSF demonstrates a certain therapeutic efficacy in treating NAFLD, the underlying mechanism of its action remains elusive.

At present, the pathogenesis and development process of NAFLD are quite complex and without comprehensive explanation. The “two-hit” hypothesis initially proposed in 1998 is considered as the classic pathogenesis of NAFLD (Day and James 1998). The first hit mainly included factors like the sedentary lifestyle and poor nutritional habits, resulting in excessive intrahepatic fat accumulation (triglyceride in main) and insulin resistance. The second hit was characterized by the overproduction of lipid-induced reactive oxidative metabolites, which in turn led to the cytokines-mediated inflammation, hepatic cell apoptosis, necrosis, fibrosis and cirrhosis in final (Berardo et al. 2020). With the deepening of research, the “two-hit” hypothesis gradually evolved into the “multiple-hit” hypothesis for a precise explanation of the etiology and pathogenesis behind NAFLD. The core idea of this theory suggested that multiple hits like nutritional factors, insulin resistance, lipotoxicity, inflammatory cascades, gut microbiota could play a role at the same time, and each factor contributed to the further progression of the disease (Buzzetti et al. 2016; Wu et al. 2022).

The TLR4/NF-κB-dependent release of inflammatory cytokines (such as TNF-α, IL-6 and IL-1β) is regarded as one of important mechanisms of NAFLD (Bessone et al. 2019). Existing studies have proved that inhibiting this pathway can improve lipid metabolism disorders in NAFLD mice and alleviate the inflammatory state of the liver (Chen et al. 2024; Deng et al. 2024), proving that inhibition of the TLR4/NF-κB signaling pathway is a potential therapeutic approach for NAFLD. COX-2 is highly expressed in inflamed tissues and it can produce inflammatory cytokines prostaglandin E2 (PGE2) from arachidonic acid (Yagami et al. 2016). Moreover, TLR4/NF-κB signaling also regulates the activation of the COX2/PGE2 axis in liver fibrosis, indicating that COX2 also plays a role in the inflammatory regulation of liver diseases (Chen et al. 2021a, b; Yang et al. 2020).

Network pharmacology employs high-throughput omics data analysis, network database retrieval and computer simulation to uncover the network relationship of herb-gene-target-disease interaction. Through delineating these intricate networks, it can predict the evaluate medicine efficacy and exact mechanisms of medicine action in disease treatment of TCM. (Shi et al. 2023; Li et al. 2022; Zheng et al. 2024). Based on this theoretical method, Wu et al. verified the mechanism of Qutan Huoxue decoction on NASH via inhibiting the SOCS1/TLR4/NF-κB inflammatory pathway with network pharmacology and in vitro experiments (Wu et al. 2023). Widespread application of HPLC-Q-TOF/MS has been employed in the structural determination of unknown chemicals in complicated mixtures via retention time, molecular mass and fragment information, which is effective in identifying probable active substances in TCM (Zhou et al. 2021; Jin et al. 2023). In addition, the integration of HPLC-Q-TOF/MS with network pharmacology has also greatly promoted the credibility of research conclusions (Zhi et al. 2023; Wang et al. 2022).

Molecular docking represents a pivotal structure-guided technique that facilitates the meticulous examination of the intricate binding interactions between protein targets and potential small-molecule ligands (Kaur et al. 2019). It was integrated synergistically within pharmacological investigations of TCM alongside network pharmacology, wherein their computationally derived predictions undergo experimental validation as a means to substantiate their findings (Liu et al. 2022; Bai et al. 2023; Jiao et al. 2021). The comprehensive advantage of network pharmacology combined with molecular docking is that it provides concepts for the investigation of complex TCM, which is progressively being used in the study of NAFLD pathogenesis and treatment (Ren et al. 2022).

Therefore, we firstly detected the therapeutic effects of WSF against NAFLD by evaluating the biochemical indices and histopathology in a high-sucrose and high-fat (HSHF) diet mice. Moreover, HPLC-Q-TOF/MS, bioinformatics in conjugation with molecular docking were applied to plumb the probable mechanism of WSF for the therapy of NAFLD in this research. Finally, we further validated the predicted pathway by immunohistochemistry, ELISA and western blot using liver tissue from the efficacy experiment in vivo. This study would offer persuasive evidence for the clinical promotion of WSF later.

## Materials and methods

### Chemical and biological reagents

Polyene Phosphatidylcholine (Essentiale, PPC) capsule was gained from Sanofi–Aventis Pharmaceutical Co., Ltd. (Beijing, China). Total cholesterol (TC), triglyceride (TG), high density lipoprotein cholesterol (HDL-c), glucose (GLU), aspartate transaminase (AST), alanine transferase (ALT) assay kits were obtained from Ningbo Medical System Biotechnology Co., Ltd. (Ningbo, China). Hematoxylin–Eosin (H&E) reagent was acquired from Shanghai Yuanye Biotechnology Co., Ltd. (Shanghai, China). Oil red O reagent was purchased from BBI Life Science Corporation (Shanghai, China). TC and TG assay kits for liver were both attained from Nanjing Jiancheng Bioengineering Institute (Nanjing, China). The ELISA kits of IL-1β and TNF-α were derived from Jiangsu Meimian Industrial Co., Ltd. (Yancheng, China). Instant immunohistochemistry kits were gained from Wuhan Boster Biological Technology Co., Ltd. (Wuhan, China). The DAB staining kit, BCA assay kit, RIPA buffer, and protein free rapid blocking buffer were got from Beyotime Biotechnology Reagent Co., Ltd. (Shanghai, China). The antibodies of COX2, IL-6 and secondary antibodies were purchased from Proteintech Group Inc. (Wuhan, China). The antibodies for TLR4, NF-κB and β-actin were acquired from Beijing Biosynthesis Biotechnology Co., Ltd. (Beijing, China), Hangzhou DiagBio Biotechnology Co., Ltd. (Hangzhou, China), and Wuhan Servicebio Technology Co., Ltd. (Wuhan, China), respectively. The enhanced chemiluminescent assay kit was attained from Nature Biosciences Ltd. (Hangzhou, China). The catalog numbers of utilized regents are included in supplementary file.

### Drug preparation

WSF was prepared by the Traditional Chinese Medicine Health Products Institute of Zhejiang University of Technology, and was then condensed to concentration of 2.853 g/mL in crude herb amounts. It was then refrigerated in 4 ℃ for later use.

### Animal experiment

ICR mice (male, n = 32) were purchased from Laboratory Animal Center of Zhejiang Academy of Medical Sciences (SYXK(Zhe)2019–002, Hangzhou, China). All the animals were reared in standardized environmental conditions characterized by a 12-h light–dark photoperiod with unrestricted access to water and food. The animal procedures were meticulously conducted in strict adherence to the Zhejiang University of Technology’s Guidelines for the Care and Use of Laboratory Animals.

Our group previously established that the ideal concentration of WSF for treating rats with glucose and lipid metabolic disorders is 14.26 g/kg (detailed data is in supplementary file). And for this study, the dosage for NAFLD mice was determined to be 28.53 g/kg based on the specific surface area method for animal dose conversion.

Considering the optimal dosage was determined and the principle of reduction in animal experiments, we used a single dosage in this study. After adaptive feeding for 1 week, a total of 32 ICR mice were randomly assigned into four groups according to their body weight with the random number method: the normal group (NC), the model group (MC), PPC administration group (PPC) and WSF administration group (WS), each consisting of 8 mice. The NC mice was fed with standard diet, while mice in the MC, PPC and WS group were all received a high-sucrose and high-fat (HSHF) diet over a period of 17 weeks. The PPC and WS mice were daily administered PPC or WSF at a dose of 273.6 mg/kg or 28.53 g/kg (i.g.) adjusted to the volume of 1 ml/100 g in accordance with the body weight, respectively. The HSHF diet, consisting of 10% lard, 10% egg yolk powder, 5% sucrose, 2% cholesterol, 0.5% sodium cholate was derived from Trophic Animal Feed High-Tech Co.,Ltd. (Nantong, China).

Upon conclusion of the experiment, mice were subjected to an overnight fasting before blood was drawn from the ocular venous plexus. Obtained blood samples were centrifuged for 10 min twice at 3600 rpm to attain serum for biochemical analysis. The mice were subsequently anesthetized (isoflurane inhalation) with removing their liver as quickly as possible. Part of liver was put in 4% paraformaldehyde tissue fixation solution for hepatic pathology and immunohistochemistry analysis, part of liver was put in ethanol absolute for liver homogenate and remaining parts were preserved at − 80 °C.

Moreover, we conducted a separate 28-day oral toxicity experiment (detailed data are provided in the supplementary file), which demonstrated that administration at 12 times the human clinical dose did not exhibit significant toxic effects on the liver or glucose and lipid metabolism in normal mice.

### Histological staining

The Hematoxylin–eosin (H&E) and Oil Red O staining procedures were conducted consistent with the methods detailed in prior literature (Lei et al. 2019). H&E staining were undergone on 4 μm paraffin-embedded tissue slices and liver pathology scores for NAFLD were decided in line with NAFLD activity score (NAS) nominated by American Association for the Study of Liver Diseases as Table 1 described (Kleiner et al. 2005). The specific procedures of tissue dehydration and the H&E staining are described in the supplementary file.

**Table 1:** Scoring standard of NAFLD activity

NAS (0–8 points)	
1. Steatosis (0–3 points)	
0	< 5%
1	5–33%
2	33–66%
3	> 66%
2. Lobular inflammation (0–3 points)	
0	No foci
1	< 2 foci in 200 × vision
2	2–4 foci in 200 × vision
3	> 4 foci in 200 × vision
3. Ballooning (0–2 points)	
0	None
1	Few
2	Many or prominent ballooning

Oil Red O staining serves as a reliable method for both the identification of lipid accumulation and the semi-quantitative assessment of hepatic steatosis. In brief, liver tissue specimens were first dehydrated in a 30% sucrose solution prior to being embedded in OCT as well as sliced into 10 μm-thick frozen sections. The 0.5% Oil Red O solution was subsequently stained on the obtained cryosections, followed by hematoxylin counterstaining on the nuclei. All H&E and Oil Red O stains were captured under the biological microscope (BX43, Olympus, Japan) and subjected to semi-analysis using the Image J software (version 1.54f). The code used for Oil Red O analysis are included in supplementary file.

### Measurement of biomarkers in serum and liver

Serum blood glucose (GLU), transaminase (AST and ALT), and lipid levels (TC, TG, and HDL-c) were determined with the respective assay reagents on the automated biochemical analyzer (HITACHI-7020, Japan). The level of serum LDL-c was further calculated by Friedwald formula (Molavi et al. 2020).

$${\text{Friedwald formula}}:{\text{ LDL}} - {\text{c }} = {\text{ TC }}{-}{\text{ HDL}} - {\text{c }} - {\text{ TG}}/{5}$$

To measure the concentrations of TC and TG in the liver, liver organs (circa 100 mg) were put into ethanol absolute (9 times in volume), homogenized for a 10% (w/v) homogenate solution and centrifuged at 2500 rpm for 10 min twice later for the acquisition of supernatant. After mixing the 2.5 μL tissue extract solution with 250 μL working solution and allowing it to stand for 10 min at 37 ℃, we measured the absorbance at a wavelength of 500 nm (TC) or 510 nm (TG), respectively. The specific concentrations of TC and TG were calculated through the standard curve.

### Qualitative identification of components in WSF

#### Sample preparation

The WSF powder was obtained using the vacuum freeze dryer (− 55 ℃, 20 Pa). Then, 3.0 g WSF powder was extracted ultrasonically (40 kHz, 300 W, likewise below) for 60 min with 30 mL methanol (chromatographic purity, likewise below) and the resultant solution was evaporated in an 85 ℃ water bath until dryness. Another 3 mL methanol was used to redissolve the residue with ultrasound for 30 min. Finally, 1.0 mL supernatant fluid underwent filtration via a 0.22 μm organic phase filter membrane with the syringe and relocated in a sampling vial for qualitative analysis.

#### HPLC-Q-TOF/MS conditions

The HPLC-Q-TOF/MS system was composed of a 1290 Infinity II liquid chromatography and a 6545XT AdvanceBio Quadruple Time-of-Flight mass spectrometry (Agilent Technologies, USA).

The chromatographic conditions were as follows: (1) Column: Welch Ultimate LP-C18 (4.6 × 250 mm, 5 μm); (2) Injection volume: 10 μL; (3) Column temperature: 30℃; (4) Flow rate: 1 mL/min; (5) Mobile phase A: water (comprising 0.1% formic acid, analytical purity), B: acetonitrile (chromatographic purity) with the changeable gradient of 0 min, 5% B; 10 min, 10% B; 40 min, 13% B; 55 min, 16% B; 65 min, 20% B; 90 min, 21% B; 100 min, 26% B; 120 min, 28% B.

The operating conditions of mass spectrometry were set as follows: (1) Ion source: Agilent jet stream electrospray ionization source (AJS-ESI); (2) Gas flow rate: 8 L/min; (3) Gas temperature: 300 ℃; (4) Sheath gas temperature: 350 ℃; (5) Nebulizer gas pressure: 35 psi; (6) Sheath gas flow rate: 11 L/min; (7) Capillary voltage: 3100 V; (8) Fragmentor voltage: 175 V; (9) Nozzle Voltage: 1000 V. Scanning with a range of m/z 50–3200 was utilized for sample mass spectrometry signal acquisition in positive and negative ion modes, respectively. All of the collected data were processed utilizing the Agilent MassHunter workstation software (version B.08.00).

### Bioinformatics analysis

#### Prediction of prospective WSF targets associated to NAFLD

The components of herbs in WSF were also collected with the TCMSP database (https://old.tcmsp-e.com/tcmsp.php, version 2.3), while the bioactive ingredients of Shanzha were identified by a supplemented literature search due to the lack in the TCMSP database (Cheng et al. 2023). The chemical components obtained from HPLC-Q-TOF/MS and database mining were sifted with a criterion of oral bioavailability (OB ≥ 30%) and drug-like properties (DL ≥ 0.18) as well as further screened through the Swiss ADME (http://www.swissadme.ch/index.php) platform with a score of “High” for gastrointestinal absorption and at least two “Yes” for drug like properties. The filtered ingredients were later transmitted to the Swiss Target Prediction platform (http://www.swisstargetprediction.ch) for protein target search with their canonical SMILES attained from PubChem database (https://pubchem.ncbi.nlm.nih.gov). The targets with a credibility value of 0 were deleted.

GeneCards (https://www.genecards.org, version 5.22.0 Build 1354), DisGENET (https://www.disgenet.org, version 24.3) and OMIM database (https://www.omim.org) were applied to obtain the NAFLD-relevant protein targets, and the keywords were set as “non-alcoholic fatty liver disease”. Targets from GeneCards database that not exceeding the median relevance scores were eliminated, and duplicate entries were consolidated to derive the set of NAFLD-related targets. Finally, the herbs-active components-targets network was constructed and visualized via Cytoscape 3.7.1 software after standardization of names with Uniprot database (https://www.uniprot.org, release 2024_05).

“Network analyzer” function in Cytoscape 3.7.1 was conducted to evaluate the overall situation of the network with topological parameters encompassing degree, average shortest path length (ASPL), betweenness centrality (BNC) and closeness centrality (CNC).

#### Coincident targets identification and protein–protein interaction (PPI) network analysis

The intersectional analysis of Venny 2.1.0 (https://bioinfogp.cnb.csic.es/tools/venny/index.html) was utilized to collect the coincident targets shared between biologically active constitutes of WSF and NAFLD-associated targets which were regarded as prospective targets of WSF to ameliorate NAFLD. The STRING database (https://string-db.org, version 12.0) was employed to build the PPI networks with constraints of a medium confidence threshold of 0.400 and homo sapiens. Subsequently, the resulting data was submitted to Cytoscape 3.7.1 for visualization purposes. Core targets were further discerned through the topological analysis of the CentiScaPe 2.2 plug-in in terms of parameters like betweenness, closeness and degree.

#### Gene ontology (GO) function and Kyoto encyclopedia of genes and genomes (KEGG) enrichment analysis

The intersecting targets derived above were uploaded into DAVID database (https://david.ncifcrf.gov, v2024q2) for KEGG and GO enrichment analysis, encompassing biological process (BP), cellular component (CC) and molecular function (MF) analysis. In the KEGG analysis, pathways with less than 0.05 *P*-values and their accordant enriched targets were selectively retained to identify crucial pathways and pivotal targets related to the impact of WSF on NAFLD. Finally, Weishengxin (http://www.bioinformatics.com.cn) was utilized for visualization purposes of the top 20 BP and KEGG pathway results according to gene counts or ratios in bubble and bar graphs.

### Molecular docking

The bioactive components with degree values ≥ 10 in the “active components-target-pathway” network were regarded as ligands in subsequent molecular docking analysis. The 2D structure of aforesaid components were obtained from the PubChem database (https://pubchem.ncbi.nlm.nih.gov), while the 3D structures with minimum energy were drawn through Chem3D software. The protein structure database Protein Data Bank database (http://www.rcsb.org) was utilized to retrieve the structural details about the core targets in inflammation related pathways with restrictions of homo sapiens, X-ray diffraction, refinement resolution ≤ 2.5 Å. The water molecules and original ligand structures in the selected proteins were removed, as well as hydrogen atoms were added by PyMoL software. Subsequently, molecular docking explorations were conducted to evaluate the optimal semi-flexible binding modes with retained rotatable bonds of the biochemicals by using the AutoDock Vina with Vina default force field and scoring function (Trott and Olson 2010).

In an effort to improve the precision of molecular docking predictions, we conducted additional flexible docking simulations. Building upon the minimum binding energy conformation derived from a semi-flexible docking protocol, we designated amino acid residues within a 5 Å sphere centered on the ligand as flexible, permitting free rotation of their side chains. AutoDock Vina was then re-employed for docking, and conformations exhibiting intramolecular hydrogen bonds were discarded.

To determine the final ranking, we calculate the sum of the rankings of the lowest binding energies of a certain molecule to all proteins firstly, and then sort the numerical values of the ranking sums of all molecules in ascending order. Ultimately, the hydrogen bond linkage with protein residues of the top two optimal biomolecules with the minimum binding energy ranking were visualized by PyMoL software.

### Immunohistochemistry (IHC) analysis of related proteins in the liver

The expression and localization of Toll-like receptor 4 (TLR4), nuclear factor-kappa B (NF-κB), cyclooxygenase 2 (COX-2) and interleukin-6 (IL-6) in the liver were determined by IHC analysis. The procedures for IHC staining were comparable to we described previously (Li et al. 2019a, b). In brief, the tissue slices were subjected to procedural deparaffinization, antigen retrieval with citrate buffer liquid (pH 6.0), incubation with appropriate primary antibodies (1:200 dilution) and HRP-conjugated secondary antibodies in turn, respectively. Subsequently, the positive expression signals were visualized under the optical microscope via DAB staining in yellow color, while the nuclei underwent counterstaining with hematoxylin. The protein expression data were subjected to semi-quantitative analysis through the computation of integrated optical density (IOD) values within the positively stained areas of microphotographs with the Image-Pro Plus software (version 6.0). The catalog and lot numbers of utilized antibodies are included in supplementary file.

#### ELISA determination of inflammation factors in the liver

The liver tissues were precisely weighed with the subsequent addition of an equivalent volume of saline solution at a 1:10 (w/v) ratio and thorough mixing. Then, the supernatants schemed for the following trails were subjected to homogenization and centrifugation at 12,000 rpm from the resultant mixtures above at 4 °C for 10 min. In final, the mouse ELISA kits of tumor necrosis factor α (TNF-α) and interleukin-1β (IL-1β) were utilized for their concentration determination in the liver as per the provided instructions.

### Western blot (WB) analysis of related proteins in the liver

In short, precisely weighted liver tissue was lysed in the RIPA buffer containing protease inhibitor and EDTA (100:1:1, v/v/v) at 4 ℃ with subsequent homogenate centrifugation for 10 min to get supernatants at 12,000 rpm for further WB analysis, prior to the concentration’s detection of total protein via the BCA assay kit. After being degenerated with loading buffer at 95 ℃, protein samples were isolated via 10% SDS-PAGE under conditions of 80 V for 30 min, followed by 120 V for 90 min and subsequently electrotransferred onto a nitrocellulose membrane in ice (200 mA with TLR4: 90 min, NF-κB and COX2: 60 min, β-actin: 40 min). Membranes were then blocked at room temperature with protein-free rapid blocking buffer for 20 min, followed by overnight incubation with respective primary antibodies (TLR4 1:1000, NF-κB 1:1000, COX-2 1:750, dilution) at 4 ℃. β-actin (1:1000, dilution) was served as the loading control. After a 1-h incubation with paired HRP-conjugated secondary antibodies, the protein bands were visualized via an enhanced chemiluminescent detection system (ChemiScope 6000, CLINX, China). The semi-quantification of gauging protein expressions was standardized against β-actin densitometry conducted with ImageJ software (version 1.53K).

### Statistical analysis

All data were presented as the average values ± standard deviation (SD). Statistical analyses entailed one-way analysis of variance (ANOVA), supplemented with Tukey’s honest significant difference (HSD) test for intergroup comparisons. A statistical significance threshold was set at *P* ≤ 0.05. All analyses of statistics were performed through an updated version of SPSS software. Diagram visualization was implemented by GraphPad Prims.

## Results

### WSF attenuated body weight and visceral fat accumulation

**Fig. 1:**
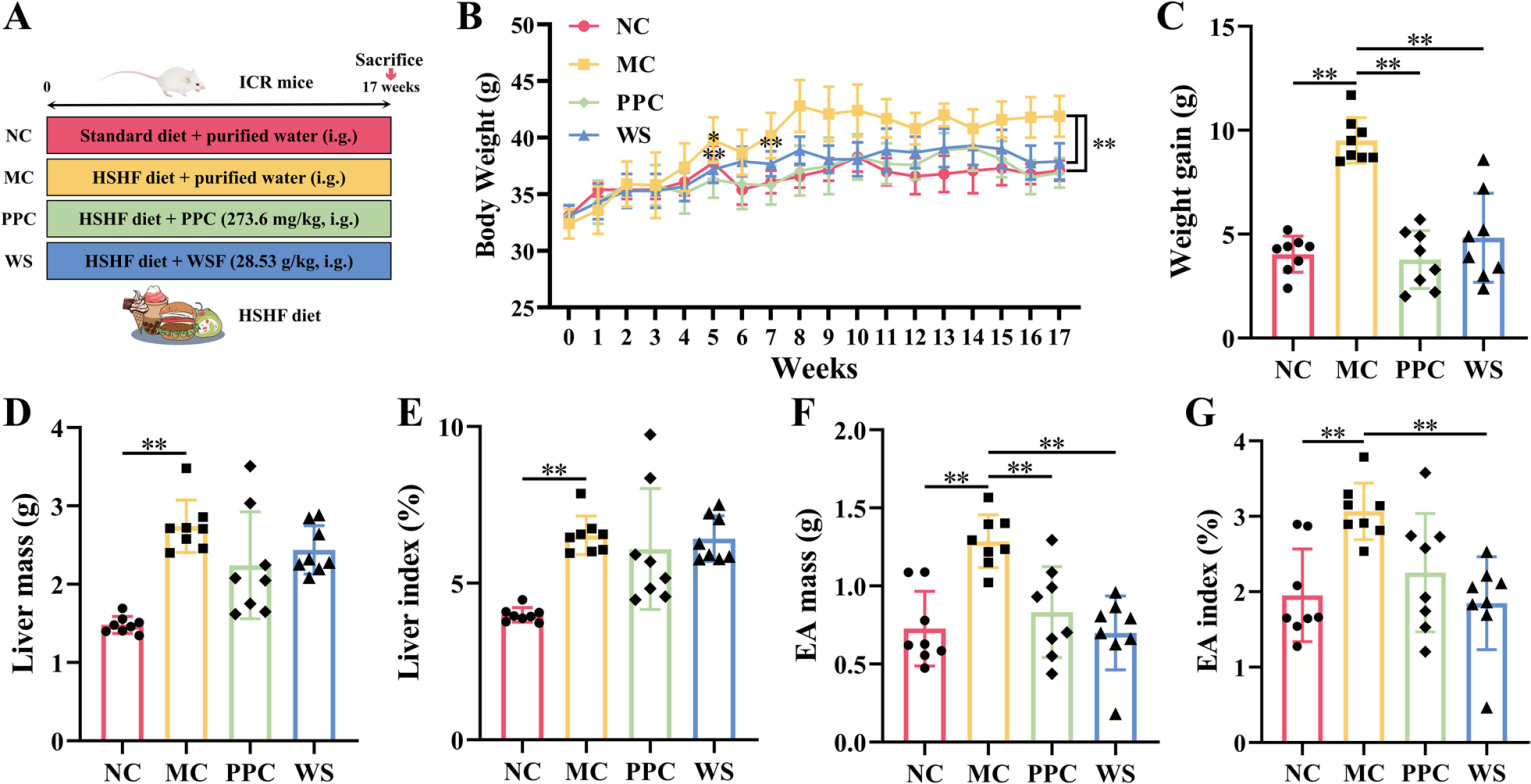
Effects of WSF on body weight and visceral fat accumulation. **A** Animal experiment procedures. **B** Body weight change during the whole experiment. **C** Weight gain. **D** Liver mass. **E** Liver index. **F** Epididymis adipose mass. **G** Epididymis adipose index. All values were presented as mean ± SD with significance markers of ^*^P < 0.05 and ^**^P < 0.01

Animal experiment procedures are show in Fig. 1A. The body weight of the MC mice increased significantly since modeling since the 5th weeks compared with the NC group (*P* < 0.05) (Fig. 1B). And after modeling for 17 weeks, the weight gain, liver mass, and epididymal adipose (EA) mass of the MC mice also increased significantly (*P* < 0.01) (Fig. 1C–E). PPC, a key component of essential phospholipids renowned for its efficacy in treating NAFLD, is instrumental in preserving hepatic cell membrane fluidity and functionality (Lu et al. 2022). Compared to the MC group, the body weight of the PPC and WS mice decreased significantly, starting from the 5th and 7th weeks of PPC and WSF administration (*P* < 0.01), respectively (Fig. 1B). Besides, the weight gain, EA mass and its index of the PPC and WS mice decreased (*P* < 0.01), while PPC has no obvious effect on EA index (Fig. 1C–G). These results indicated that WSF treatment could reverse abnormal weight gain and visceral fat accumulation caused by the HSHF diet.

### WSF improved liver function and pathological lesions

**Fig. 2:**
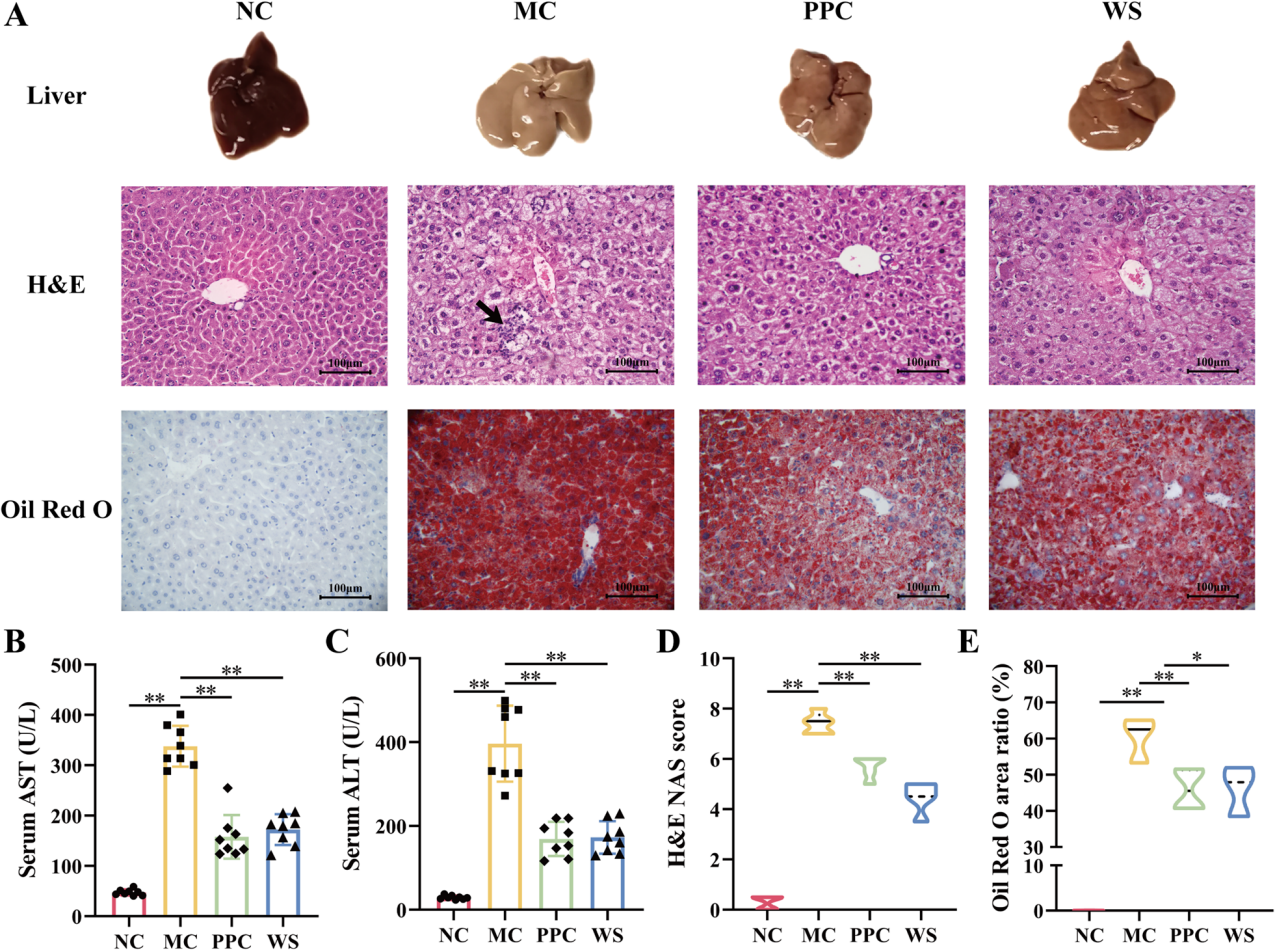
Effects of WSF on liver function and hepatic pathology. **A** Representative graphs of morphology and pathological changes in liver (H&E 400 × ; Oil Red O 400 ×). **B** Serum AST. **C** Serum ALT. **D** NAFLD activity scores. **E** Oil Red O staining area ratio. All values were presented as mean ± SD with significance markers of ^*^P < 0.05 and ^**^P < 0.01

After modeling for 17 weeks of the HSHF diet, the serum levels of AST and ALT in the MC mice increased significantly compared with the NC group (*P* < 0.01) (Fig. 2B, C). The H&E and Oil Red O staining demonstrated an increase of inflammatory foci (indicated by arrowheads), extensive hepatocyte ballooning lesions, and aberrant lipid accumulation in the liver of NAFLD mice (Fig. 2A, E). Besides, the NAS score of the MC mice also increased significantly (*P* < 0.01) (Fig. 2D), demonstrating that the normal physiological structure of the liver was severely damaged. Compared with the MC group, PPC and WSF could lessened the levels of serum AST and ALT significantly (*P* < 0.01) (Fig. 2B, C). Meanwhile, the inflammatory cell infiltration was reduced and other pathological changes of the liver were reversed as attested by H&E and Oil Red O staining (Fig. 2A). In addition, the NAS score and Oil Red O area ratio decreased in various degrees (*P* < 0.05, 0.01) (Fig. 2D, E). Collectively, these results showing that WSF improved liver function and attenuated pathological lesions of inflammatory and steatosis.

### WSF ameliorated lipid metabolism

**Fig. 3:**
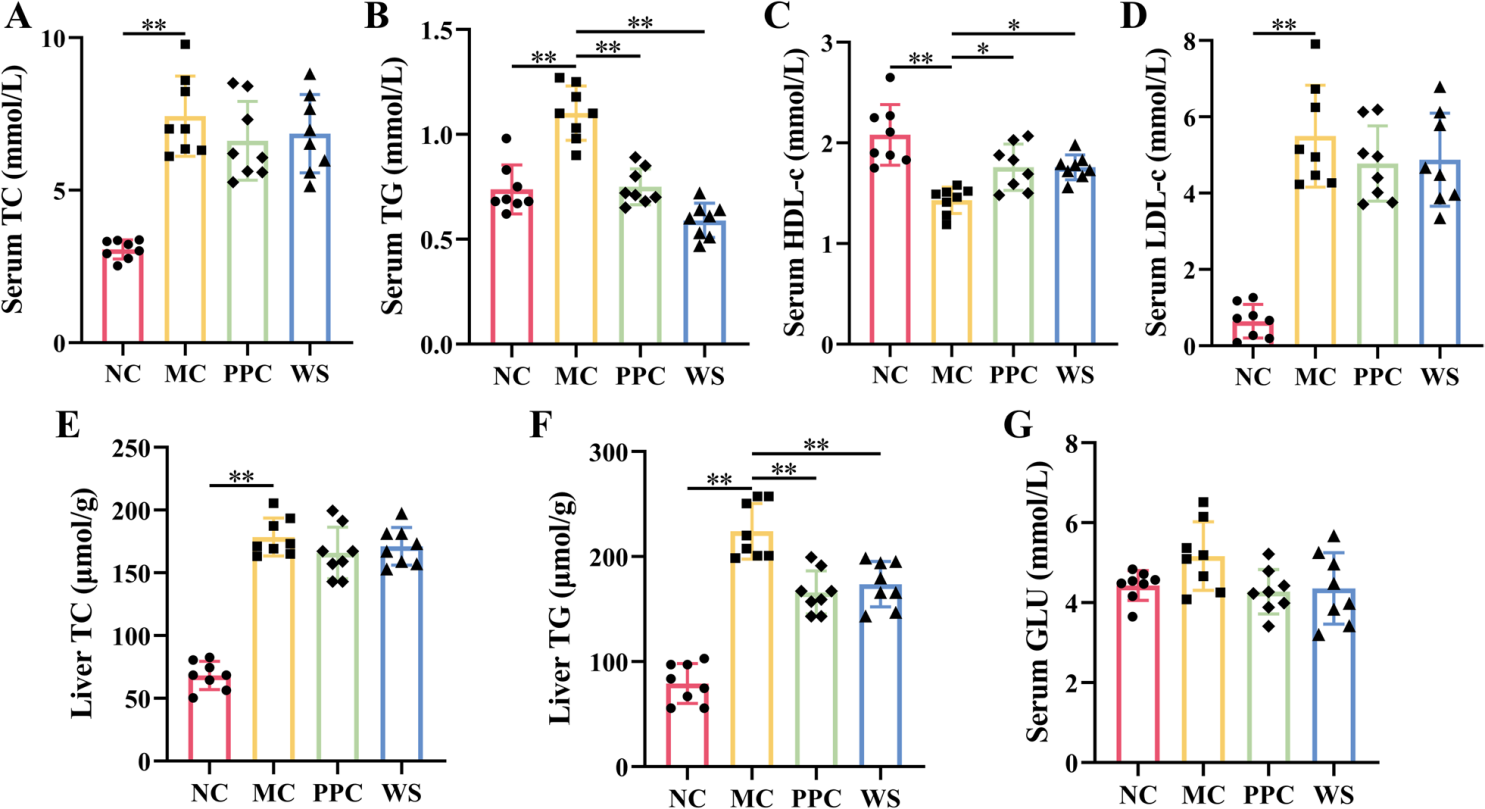
Effects of WSF on glycolipid metabolism. **A**–**D** Levels of TC, TG, HDL-c, and LDL-c in serum. **E**, **F** Levels of TC and TG in liver. **G** Levels of GLU in serum. All values were presented as mean ± SD with significance markers of ^*^P < 0.05 and ^**^P < 0.01

In divergence from the NC group, the levels of serum TC, TG, LDL-c as well as liver TC, TG in the MC mice increased significantly (*P* < 0.01) (Fig. 3A, B, D-F), while serum HDL-c levels decreased obviously (*P* < 0.01) (Fig. 3C) after modeling for 17 weeks, suggesting that there might be abnormal lipid metabolism in HSHF diet-induced NAFLD mice. Compared with the MC group, PPC and WSF treatment could suppress the increase of serum TG and liver TG and the decrease of serum HDL-c in various degrees (*P* < 0.05, 0.01), but had no effect on serum TC and LDL-c (Fig. 3A–F). Moreover, we observed that after 17 weeks of modeling, there was a trend towards elevated serum GLU levels in MC mice (without significant difference), whereas administration of PPC and WSF could normalize the serum GLU levels. These results suggested that WSF could ameliorated and lipid metabolism disorder induced by NAFLD through regulating the serum levels of TG and HDL-c and TG accumulation in the liver.

### Identification of phytochemical components in WSF

**Fig. 4:**
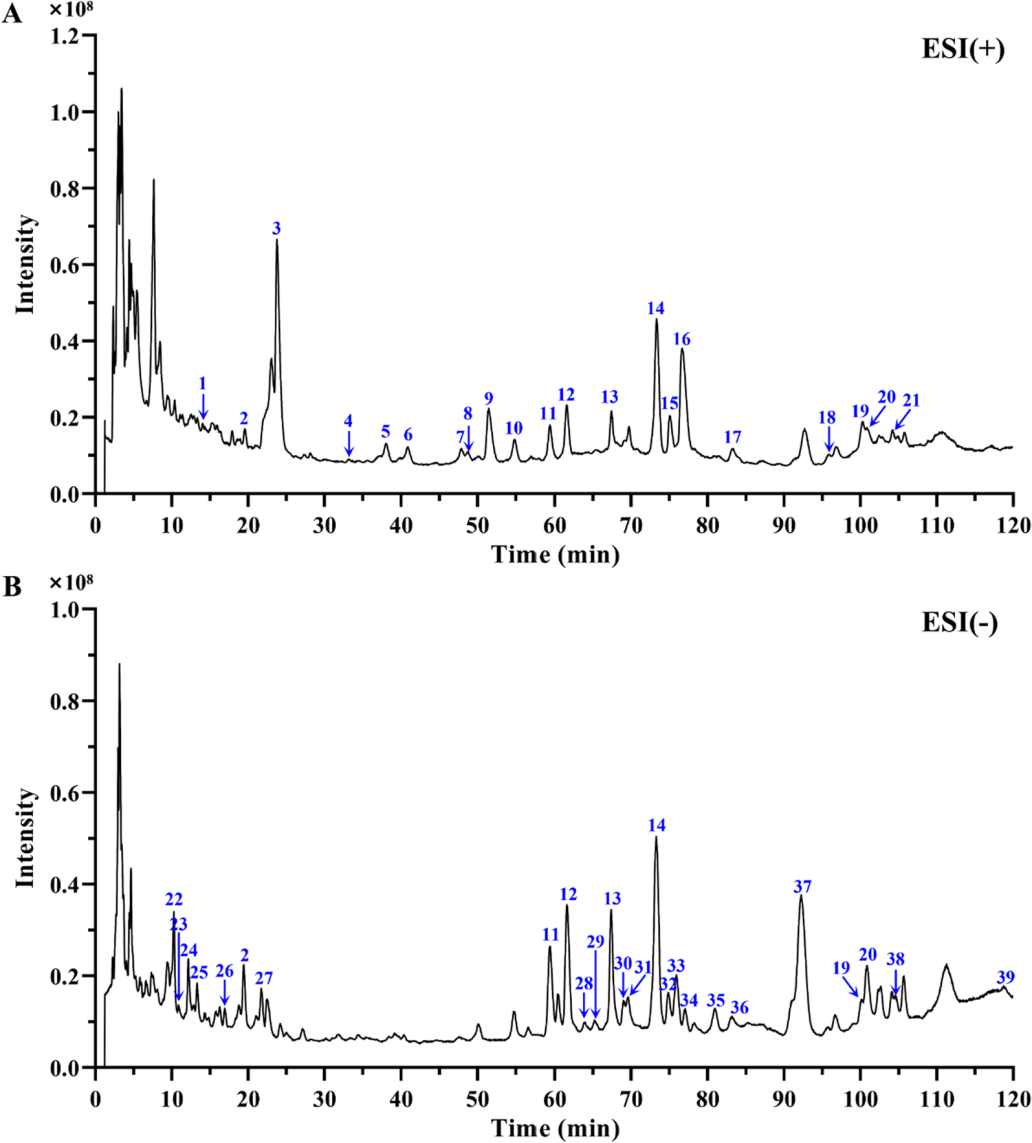
The total ion chromatogram (TIC) profiles in positive (**A**) and negative ion mode (**B**). Peaks 1–39 correlate with the compounds enumerated in Table 2

The Agilent HPLC-Q-TOF/MS system was exploited to evaluate phytochemical components of WSF, and total ion chromatogram profiles were acquired via full scan under both positive and negative electrospray ionization modes (Fig. 4). In comparison with the HMDB, MassBank, TCMSP databases and data from related literature searched from CNKI, Wanfang and VIP databases, 39 potential compounds were tentatively recognized in accordance with their retention time (RT), molecular formulas, differences in theoretical and observed quasi-molecular ion mass (m/z), errors in ppm, scores of Agilent Masshunter and MS/MS fragment ion circumstances in total. Wherein, 7 kinds of mutual compounds were identified simultaneously in positive and negative modes. The majority of the detected components were attributed to flavonoids (including flavone glycosides), alkaloids, phenylpropanoids (including coumarins and lignans) and phenolic acids. The specific information of components identified tentatively in WSF was listed in Table 2.

**Table 2:** Identification of chemical compounds in WSF via HPLC-Q-TOF/MS

No	RT (min)	Putative compounds	Molecular formula	Observed m/z	Theoretical m/z	Error (ppm)	Score	ESI mode	CAS number
1	14.021	Higenamine	C_16_H_17_NO_3_	272.1273	271.1208	3.59	94.64	+	5843-65-2
2	19.589	Chlorogenic acid	C_16_H_18_O_9_	355.1011	354.0951	3.85	91.21	+	327-97-9
	19.404	Chlorogenic acid	C_16_H_18_O_9_	353.0879	354.0951	0.20	97.67	–	327-97-9
3	23.777	Sinapine	C_16_H_24_NO_5_	310.1642	310.1654	2.32	95.86	+	18696-26-9
4	33.151	Phenprobamate	C_10_H_13_NO_2_	180.1008	179.0946	6.05	93.81	+	673-31-4
5	38.053	(-)-Armepavine	C_19_H_23_NO_3_	314.1745	313.1678	2.20	96.73	+	524-20-9
6	40.895	(-)-N-Methylcoclaurine	C_18_H_21_NO_3_	300.1589	299.1521	2.24	95.60	+	5096-70-8
7	47.909	Asimilobine	C_17_H_17_NO_2_	268.1323	267.1259	3.91	93.42	+	6871-21-2
8	48.757	Coclaurine	C_17_H_19_NO_3_	286.1435	285.1365	1.71	97.00	+	486-39-5
9	51.466	N-nornuciferine	C_18_H_19_NO_2_	282.1482	281.1416	2.61	97.28	+	3153-55-7
10	54.823	Calycosin 7-galactoside	C_22_H_22_O_10_	447.1280	446.1213	1.77	96.77	+	114272-30-9
11	59.310	Rutin	C_27_H_30_O_16_	611.1598	610.1534	2.00	94.97	+	153-18-4
	59.310	Rutin	C_27_H_30_O_16_	609.1455	610.1534	1.28	97.92	–	153-18-4
12	61.570	Quercituron	C_21_H_18_O_13_	479.0810	478.0747	2.56	95.12	+	22688-79-5
	61.652	Quercituron	C_21_H_18_O_13_	477.0671	478.0747	1.09	98.32	–	22688-79-5
13	67.404	Naringin	C_27_H_32_O_14_	581.1857	580.1792	1.90	96.19	+	10236-47-2
	67.369	Naringin	C_27_H_32_O_14_	579.1712	580.1792	1.57	97.49	–	10236-47-2
14	73.321	Hesperidin	C_28_H_34_O_15_	611.1960	610.1898	1.96	96.28	+	520-26-3
	73.269	Hesperidin	C_28_H_34_O_15_	609.1818	610.1898	1.48	97.56	–	520-26-3
15	75.082	O-nornuciferine	C_18_H_19_NO_2_	282.1482	281.1416	2.61	97.28	+	3153-55-7
16	76.695	(-)-Nuciferine	C_19_H_21_NO_2_	296.1638	295.1572	3.23	95.11	+	475-83-2
17	83.243	Ononin	C_22_H_22_O_9_	431.1322	430.1264	3.83	89.01	+	486-62-4
18	95.807	Methylnissolin	C_17_H_16_O_5_	301.1055	300.0998	5.57	87.22	+	73340-41-7
19	100.261	Wogonin	C_16_H_12_O_5_	285.0750	284.0685	3.14	95.60	+	632-85-9
	100.110	Wogonin	C_16_H_12_O_5_	283.0613	284.0685	0.26	97.79	–	632-85-9
20	100.943	Quercetin	C_15_H_10_O_7_	303.0492	302.0427	2.89	95.01	+	117-39-5
	100.841	Quercetin	C_15_H_10_O_7_	301.0353	302.0427	0.62	98.27	–	117-39-5
21	104.200	Poncirin	C_28_H_34_O_14_	595.2003	594.1949	3.52	88.63	+	14941-08-3
22	10.280	Danshensu	C_9_H_10_O_5_	197.0457	198.0528	− 0.65	98.48	–	42085-50-7
23	10.895	Vanillic acid	C_8_H_8_O_4_	167.0351	168.0423	− 0.48	98.74	–	121-34-6
24	12.161	Protocatechuic acid	C_7_H_6_O_4_	153.0195	154.0266	− 0.82	86.79	–	99-50-3
25	13.305	Neochlorogenic acid	C_16_H_18_O_9_	353.0877	354.0951	0.80	97.77	–	906-33-2
26	16.928	Protocatechualdehyde	C_7_H_6_O_3_	137.0246	138.0317	− 0.73	99.29	–	139-85-5
27	21.731	Cryptochlorogenic acid	C_16_H_18_O_9_	353.0877	354.0951	0.65	97.38	–	905-99-7
28	63.845	Triphasiol	C_19_H_24_O_6_	347.1493	348.1573	2.48	94.09	–	81445-98-9
29	65.241	Carinol	C_20_H_26_O_7_	377.1592	378.1679	4.04	92.22	–	58139-12-1
30	68.998	Azelaic acid	C_9_H_16_O_4_	187.0977	188.1049	− 0.12	98.67	–	123-99-9
31	69.612	Astragalin	C_21_H_20_O_11_	447.0921	448.1006	2.55	44.64	–	480-10-4
32	74.831	Paramiltioic acid	C_19_H_24_O_5_	331.1549	332.1624	0.91	98.82	–	140396-85-6
33	75.928	Rosmarinic acid	C_18_H_16_O_8_	359.0774	360.0845	0.19	98.15	–	20283-92-5
34	76.992	Marmin	C_19_H_24_O_5_	331.1551	332.1624	0.55	98.30	–	14957-38-1
35	80.964	Salvianolic acid A	C_26_H_22_O_10_	493.1145	494.1213	0.16	96.16	–	96574-01-5
36	84.687	Isorhamnetin	C_16_H_12_O_7_	315.0516	316.0583	− 0.84	96.37	–	480-19-3
37	92.215	Salvianolic acid B	C_36_H_30_O_16_	717.1455	718.1534	0.99	97.51	–	121521-90-2
38	104.564	Monomethyl lithospermate	C_28_H_24_O_12_	551.1190	552.1268	1.69	95.46	–	933054-33-2
39	118.009	Ombuin	C_17_H_14_O_7_	329.0670	330.0740	− 0.28	97.70	–	552-54-5

### Bioinformatics-based analysis

#### Acquisition of WSF component targets and NAFLD disease targets

**Fig. 5:**
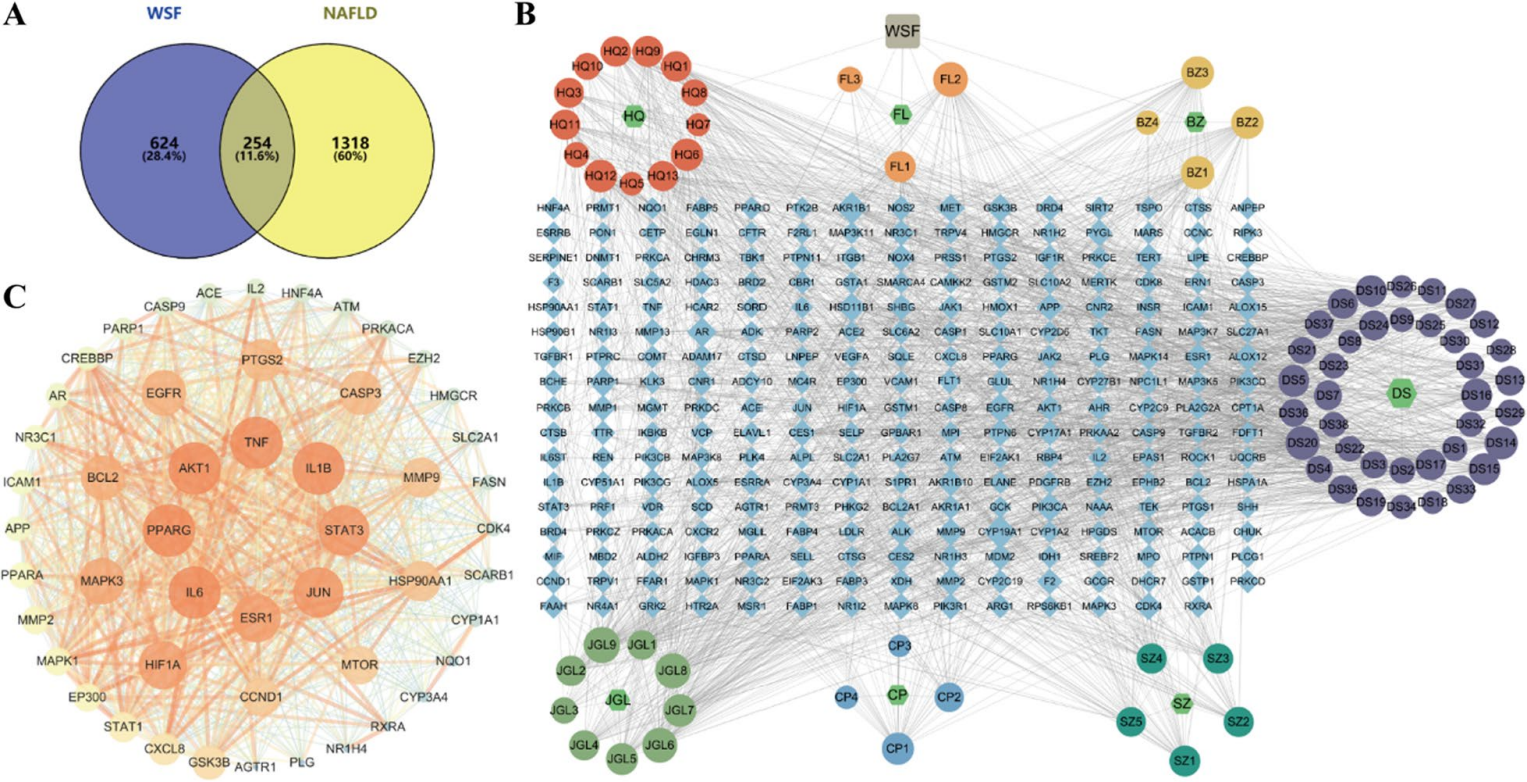
Analysis of core components and proteins for WSF on NAFLD treatment. **A** Venn diagram of targets. **B** Herbs-active components-targets network. **C** PPI network of core targets possessing the highest 50-degree values

A total of 72 chemical components from WSF were acquired from the HPLC-Q-TOF/MS results, TCMSP database and literature search after Swiss ADME platform screening. Subsequently, 878 protein targets for the 72 active ingredients present in WSF were identified by using the Swiss Target Prediction platform after removing repetitive targets with their canonical SMILES. After deduplication, a total of 1573 unique NAFLD-related targets were compiled from a combination of the GeneCards, DisGENET and OMIM databases, which were used as disease targets of NAFLD for later analysis. The Venny 2.1.0 website was further subjected to coincide targets associated with the WSF with the NAFLD disease-related targets, yielding 254 shared targets in total as prospective therapeutic targets for the NAFLD treatment by the WSF (Fig. 5A).

#### Construction of the herbs-active components-targets network map

Cytoscape 3.7.1 was utilized to construct an interaction map of the herbs-active components-targets network, consisting of 338 nodes and 2037 edges (Fig. 5B). The nodes in the network were distinguished by size, which had a positive correlation with the degree values. The map was further subjected to additional topological analysis across “Network analyzer” tool. Topological results implied that the centralization of the network is 0.135 and the heterogeneity is 1.033, while the average CNC is 0.35, showing that some nodes in the network were more concentrated and contributive than others. The average degree of the network is 12.05, and there are 63 component nodes and 55 target nodes above this value. The gypentonoside A_qt node has the highest degree value with connections to 56 targets, and the target CYP19A1 is connected to 46 components of WSF. It is indicated that bioactive components could interact with singular or multiple targets, while various components might also share common targets. Collectively, these insights propose a multi-component and multi-target mode of action for WSF in eliciting complex pathophysiological alterations pertinent to NAFLD therapy. Details of components in WSF that ranked within the top 10 based on their degree values were listed in Table 3.

**Table 3:** The information of components in WSF possessing the highest 10-degree values

Component name	Molecular formula	2D structure	Degree	ASPL	BNC	CNC
Gypentonoside A_qt	C_30_H_48_O_4_		57	2.448	0.045	0.408
Gypenoside XXVIII_qt	C_27_H_44_O_3_		53	2.478	0.037	0.404
Gypenoside XXXV_qt	C_29_H_48_O_3_		52	2.484	0.031	0.403
Cerevisterol	C_28_H_46_O_3_		51	2.531	0.052	0.395
Danshenol A	C_21_H_20_O_4_		48	2.555	0.035	0.391
12-senecioyl-2E,8E,10E-atractylentriol	C_19_H_22_O_4_		45	2.537	0.040	0.394
Przewaquinone c	C_18_H_16_O_4_		45	2.519	0.041	0.397
(3R)-3-(2-hydroxy-3,4-dimethoxyphenyl)chroman-7-ol	C_17_H_18_O_5_		43	2.596	0.024	0.385
14-acetyl-12-senecioyl-2E,8Z,10E-atractylentriol	C_20_H_22_O_5_		43	2.543	0.032	0.393
Gypenoside XXVII_qt	C_27_H_46_O_3_		43	2.531	0.030	0.395

#### Construction of the PPI network map

To further investigate the therapeutic mechanisms of WSF on NAFLD, PPI analysis of the coincident targets was implemented with the STRING database. The original PPI network comprised 253 nodes interconnected by 4943 edges with a degree value of 39.075 on average and a clustering coefficient of 0.547 on average, which characterized the intricate protein interaction collectively. Utilizing the CentiScaPe 2.2 plugin for topological analysis, the processed PPI network was subsequently visualized through Cytoscape 3.7.1 software (Fig. 5C), employing stringent selection criteria of degree > 39.075, betweenness centrality > 251.65, and closeness centrality > 0.002 to discern top 50 key genes. The brighter color and larger size of circles were positive correlation with the degree values, while the thicker width and brighter color of edges were positive correlation with the combined score. In final, a total of 50 core targets with 873 edges were screened out, and TNF (162), IL6 (157), AKT1 (157), IL1B (142), and PPARG (136) were the top 5 targets according to their degree values, and PTGS2 also has a relatively high degree value of 109, which were all considered as the key anti-NAFLD targets of WSF.

#### GO and KEGG analysis

**Fig. 6:**
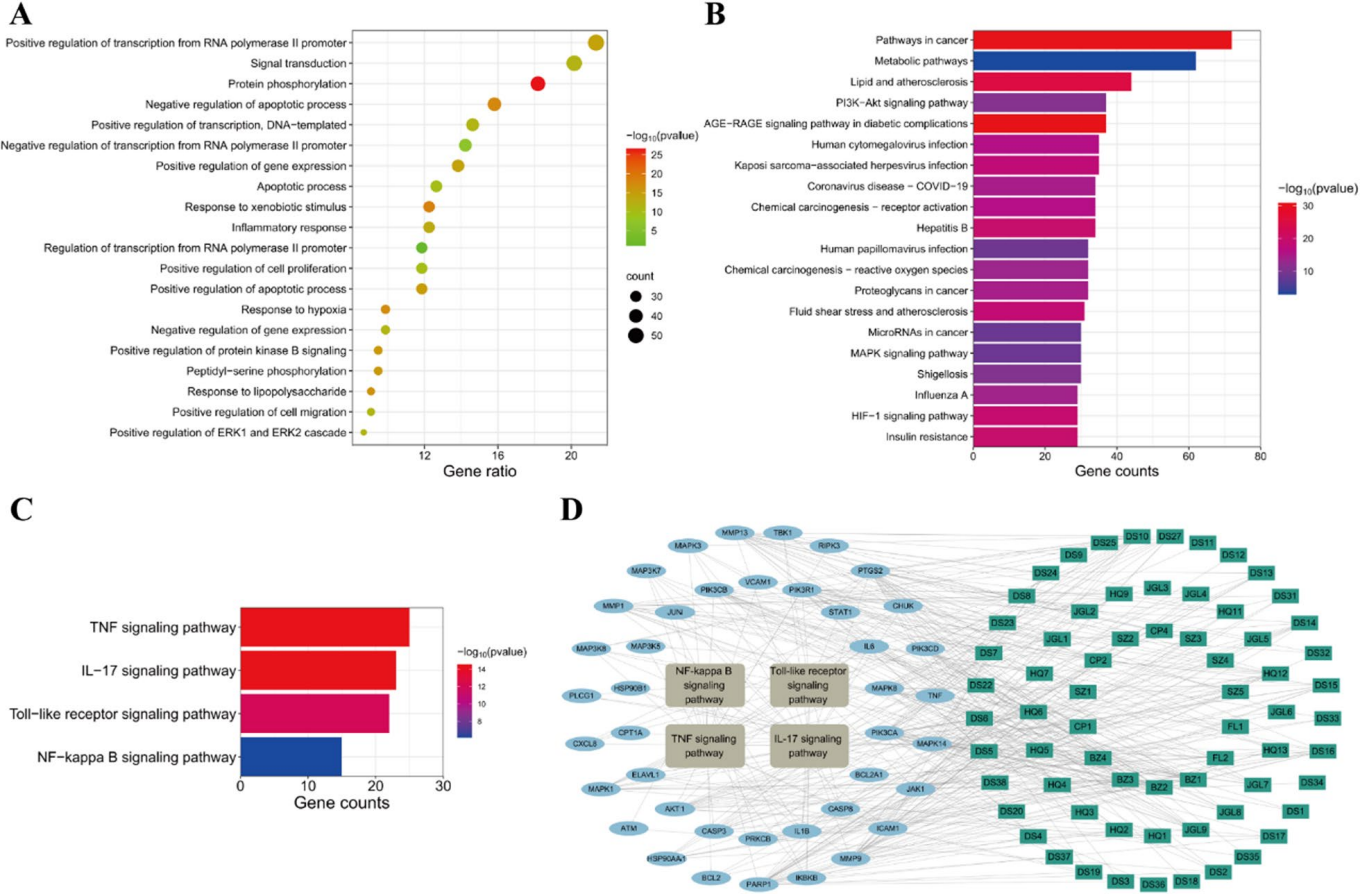
Enrichment analysis for WSF on NAFLD treatment. **A** BP results of GO analysis (top 20). **B** KEGG pathway analysis results (top 20). **C** Analytical results of selected pathways. **D** Active components-target-pathway network of WSF on treating NAFLD

GO analysis of the coincident targets mutual to WSF and NAFLD was conducted via the DAVID database. The outcomes of GO analysis disclosed that BP-related alterations were primarily in linkage with 882 functional annotations, comprising positive regulation of transcription from RNA polymerase II promoter, signal transduction, protein phosphorylation, inflammatory response (rank 10), and the response to lipopolysaccharide (LPS, rank 13), etc. (Fig. 6A). Wherein, LPS constitutes a primary composition of the outer membrane in Gram-negative bacterial cell walls and could participate in NAFLD inflammatory progression via activation of the gut-liver axis LPS/TLR4/NF-κB pathway (Han et al. 2022). Besides, alterations related to CC primarily encompassed 97 functional annotations, with a substantial focus on locations of the cytosol, cytoplasm and plasma membrane etc. And the majority of alterations related to MF were predominantly characterized by 208 functional annotations, encompassing protein binding, identical protein binding and ATP binding, etc. The CC and MF outcomes of GO analysis were not visualized in this article.

The coincident targets between WSF and NAFLD were also submitted to the DAVID database for an in-depth analysis of the KEGG, and the 156 related pathways were screened with a criterion of *P* < 0.01. The enrichment bar chart of the top 20 key pathways was drawn according to gene counts using Weishengxin website (Fig. 6B). Wherein, we found that pathways in cancer, metabolic pathways and lipid and atherosclerosis have the top 3 largest gene counts.

In accordance with PPI analysis results, we discovered that core proteins like TNF, IL6, IL1B and PTGS2 (COX2) were canonical pro-inflammatory cytokines. Moreover, the BP results also revealed the potential of WSF to ameliorate NAFLD by modulating inflammatory responses. Therefore, we conducted an in-depth exploration of the KEGG analysis results and found that inflammation related pathways like TNF, IL-17, Toll-like receptor and NF-kappa B signaling pathway were also involved in enrichment pathways with gene counts ≥ 15 (Fig. 6C). These selected 4 pathways (in brown gray) involved 41 related targets (in blue) and 67 chemical components (in green) with 112 nodes and 408 edges (Fig. 6D). In conclusion, the combination results of KEGG and PPI analysis indicated that WSF might act on NAFLD through proteins in inflammation related pathways above, which would be validated with further experiments later.

### Molecular docking

**Fig. 7:**
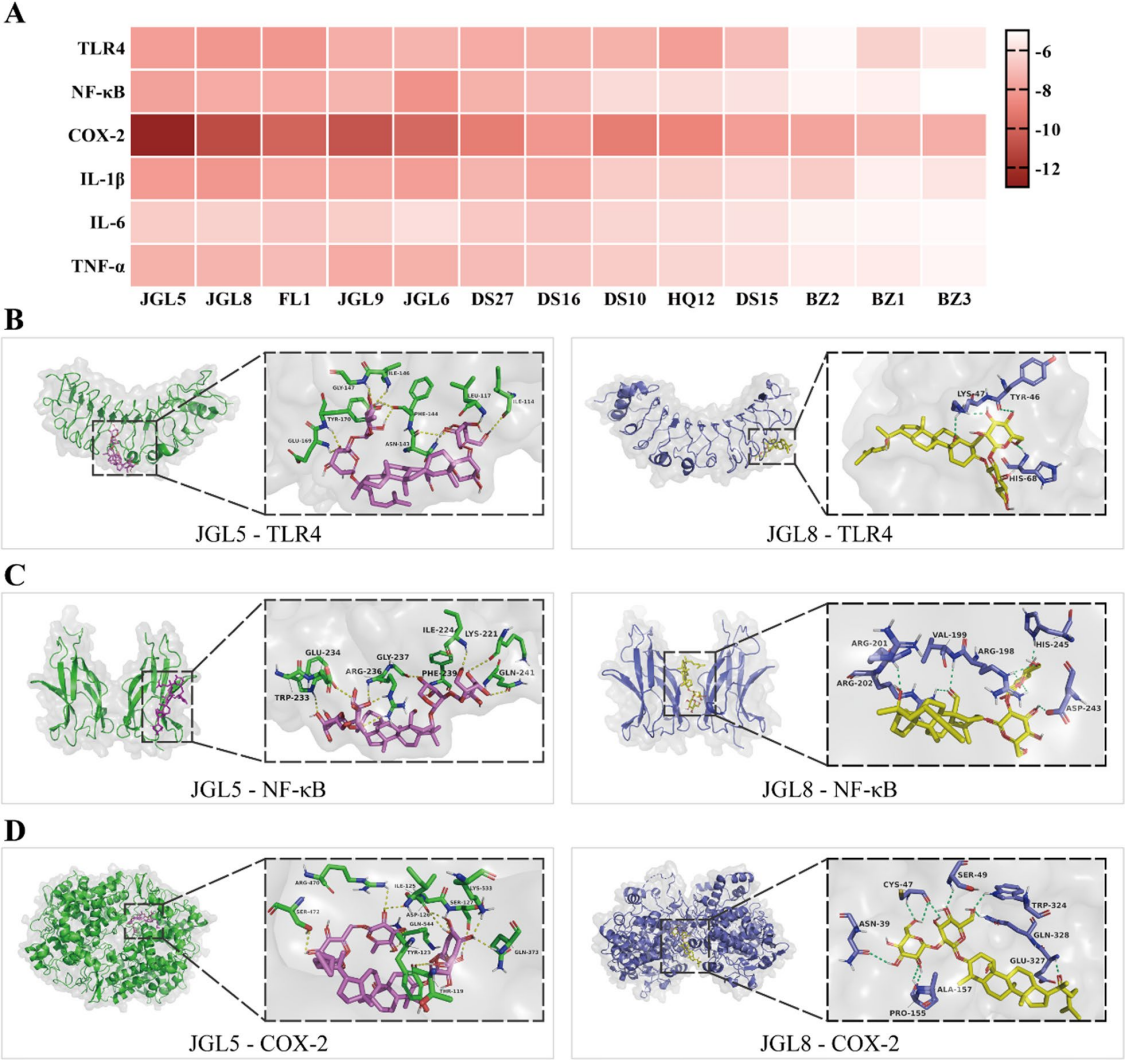
Molecular docking results of ingredients in WSF with pivotal proteins. **A** Heat map in accordance with minimum binding energies. **B**–**D** The docking conformation of JGL5 and JGL8 with TLR4, NF-κB and COX-2

For the further affinity verification, molecular docking was carried out of the core proteins in inflammation related pathways (TLR4, NF-κB, COX-2, IL-1β, IL-6, and TNF-α) with 13 active components of degree values ≥ 10 in the “active components-target-pathway” network. The respective minimum binding energies between various active components with their CAS numbers and proteins with their PDB IDs were listed in Table 4. Furthermore, a heat map was constructed according to the minimum binding energy for intuitive visualization purpose (Fig. 7A). The stability of the ligand-receptor binding was inversely proportional to the binding energy, which indicated that a more stable interaction resulted in lower binding energy. It is considered to have a good affinity between ligands and receptors if the binding energy is below − 5.0 kcal/mol (Jiang et al. 2022).

**Table 4:** The respective minimum binding energy of components in WSF with core proteins

Active components	Minimum binding energy (kcal/mol)					
	TLR4 (2Z62)	NF-κB (1MY7)	COX-2 (5F19)	IL-1β (4NI7)	IL-6 (5R8E)	TNF-α (5UUI)
JGL5 (90058-55-2)	− 8.0	− 7.9	− 12.7	− 8.1	− 6.6	− 7.4
JGL8 (81474-80-8)	− 8.2	− 7.6	− 11.0	− 8.2	− 6.4	− 7.3
FL1 (176390-66-2)	− 8.2	− 7.6	− 10.1	− 7.7	− 6.8	− 7.1
JGL9 (187277-03-8)	− 7.5	− 7.3	− 10.7	− 7.7	− 6.5	− 7.6
JGL6 (90058-54-1)	− 7.3	− 8.4	− 9.9	− 8.0	− 6.0	− 7.4
DS27 (2237283-20-2)	− 7.6	− 7.4	− 9.1	− 7.3	− 6.7	− 7.1
DS16 (142694-58-4)	− 7.4	− 7.1	− 8.2	− 7.7	− 6.8	− 6.8
DS10 (135040-83-4)	− 7.4	− 6.1	− 9.1	− 6.6	− 6.3	− 6.6
HQ12 (64474-51-7)	− 8.0	− 6.1	− 8.8	− 6.5	− 6.1	− 6.3
DS15 (515-03-7)	− 7.1	− 5.9	− 8.0	− 6.2	− 5.9	− 6.0
BZ2 (113269-37-7)	− 5.2	− 5.3	− 7.8	− 6.6	− 5.4	− 5.6
BZ1 (113269-39-9)	− 6.4	− 5.5	− 7.4	− 5.5	− 5.3	− 5.6
BZ3 (113269-36-6)	− 5.7	− 5.0	− 7.5	− 5.8	− 5.2	− 5.3

Our results implied that all the minimum binding energies were no more than -5.0 kcal/mol with a value of − 7.17 kcal/mol on average, which indicated that most of the selected chemical components of WSF were conjugated tightly with pivotal proteins in inflammation related pathways. These data further proved that WSF might exert pharmacological effects against NAFLD by mediating the activities of inflammatory related proteins of the TLR4/NF-κB/COX-2 pathway at the molecular conformation level. Besides, we found that JGL5 (gypenoside XXXVI), JGL8 (gypenoside XXVIII), FL1 (16α-hydroxydehydrotrametenolic acid), JGL9 (gypentonoside·A) and JGL6 (gypenoside XXXV) have lower minimum binding energies to almost all core targets, indicating that they might be the core components of WSF in NAFLD therapy. Representative simulation figures of core protein-active component docking patterns were drawn by PyMoL software with links between certain binding residue positions and hydrogen bonds (Fig. 7B–D).

### Effects of WSF on the critical protein expressions and inflammatory factor levels in the liver

**Fig. 8:**
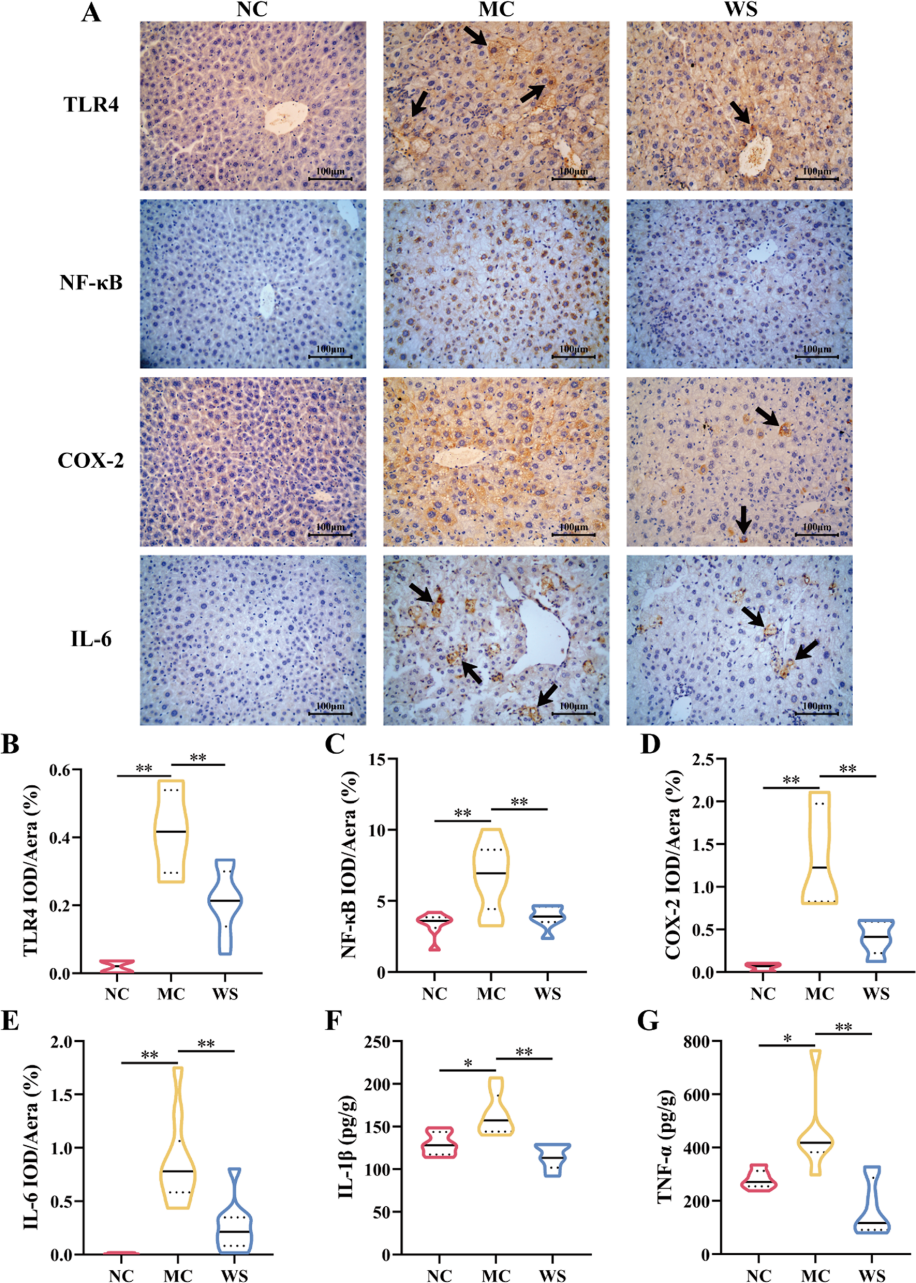
Effects of WSF on liver inflammation via IHC and ELISA analysis. **A** IHC figures at the magnification of 400 × . **B**–**E** Expression levels of TLR4, NF-κB, COX-2 and IL-6 in liver quantified by IHC assays. **F**–**G** Expression levels of IL-1β and TNF-α in liver quantified by ELISA assays. Black arrows refer to the positive expression sites of specific proteins. All values were presented as mean ± SD with significance markers of ^*^P < 0.05 and ^**^P < 0.01

**Fig. 9:**
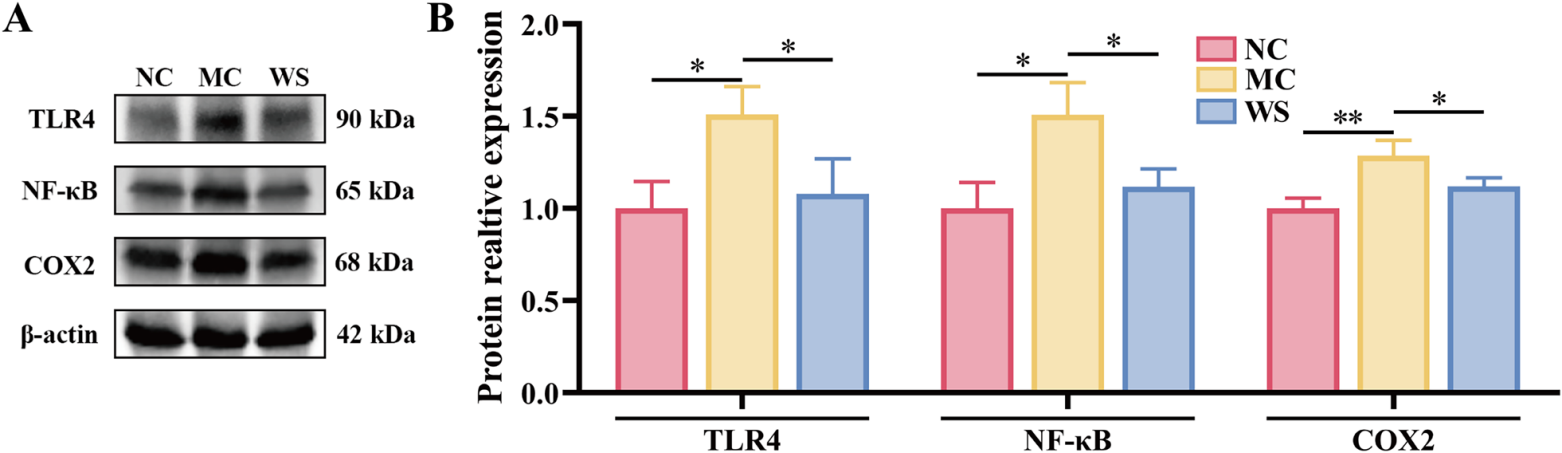
Effects of WSF on hepatic inflammation via WB analysis. **A** Representative figures of WB. **B** Relative expression levels of TLR4, NF-κB and COX-2 to β-actin in the liver quantified by WB assays. All values were presented as mean ± SD with significance markers of ^*^P < 0.05 and ^**^P < 0.01.

To further validate the anti-NAFLD effect of WSF across the inflammatory TLR4/NF-κB/COX-2 pathway in diet-induced mice, IHC (Fig. 8A), WB (Fig. 9A) and ELISA analyses of pivotal proteins and inflammation factors in the liver were performed. Compared to the NC group, the HSHF diet would induce an increase in levels of TLR4, NF-κB and COX-2 expression in NAFLD mice livers (*P* < 0.05, 0.01) (Fig. 8B–D, Fig. 9B) and promote the release of inflammation factors like IL-1β, IL-6 and TNF-α (*P* < 0.05, 0.01) (Fig. 8E–G). In contrast, intervention with WSF for 17 weeks could result in the diminution expression of TLR4, NF-κB and COX-2 (*P* < 0.05, 0.01) (Fig. 8B–D, Fig. 9B) with reversed pro-inflammatory cytokine levels in the liver (*P* < 0.01) (Fig. 8E–G). These findings highlighted that WSF treatment indeed attenuated the NAFLD and inflammation in the liver across the TLR4/NF-κB/COX-2 pathway, consistent with the results obtained above by the bioinformatics and molecular docking.

## Discussion

NAFLD refers to a clinical syndrome pathologically characterized by inordinate intracellular lipid deposition in the liver due to factors other than alcohol and other well-defined hepatocyte-damaging elements, with a spectrum of diseases including NAFL, NASH, hepatic fibrosis, cirrhosis, or even hepatocellular carcinoma (HCC) (Chalasani et al. 2018). Nowadays, the NAFLD incidence of the population in Asian countries is around 27.4%, with a continuous upward and younger trend (Fan et al. 2017; Anderson et al. 2015). NAFLD in children is characterized by rapid progression, 25.0%-50.0% have developed NASH among confirmed cases of NAFLD in children, and 10.0%-25.0% have developed liver fibrosis as reported (Goyal and Schwimmer 2016). However, current used drugs mainly focused on weight loss, metabolism regulation, antioxidation and liver protection, and long-term application might induce increased risk of cancer, hemorrhagic stroke and symptomatic heart failure (Hsu et al. 2016). Therefore, there is still a lack of efficacious hepatoprotective medicine with few side effects, which seriously lowers the life quality of NAFLD patients of all ages.

TCM formulas often contain a variety of herbs with a myriad of active ingredients that could holistically modulate the intricate pathogenic network of NAFLD, as proven by long-term safe medication practices (Sun et al. 2021). In TCM theories, NAFLD was supposed to be a syndrome of deficient in origin and excessive in superficiality. Additionally, spleen deficiency was considered as Ben (primary aspect), while pathogenic factors like blood stasis and phlegm-dampness were Biao (secondary aspects) (Zhang et al. 2010). *Synopsis of the Golden Chamber* noted that “treating liver by nourishing spleen”, indicating that if the transportation function of spleen was normal, qi and blood would be harmonized, and liver diseases could be cured as soon as possible (Wei et al. 2015). WSF was developed under the guidance of Prof. Wang Kun-Gen with the efficacity of “invigorating the spleen and benefiting the stomach, clearing heat and promoting diuresis”, which fits in with the above TCM theories of NAFLD treatment. Years of clinical experience have also proved that WSF performs well in managing metabolic diseases like hyperlipidemia and severe fatty liver (Shen et al. 2018). In summary, WSF has the potency for NAFLD prevention and treatment, but its modes of action need further systematic investigation. Therefore, we detect the pharmacological effects and relevant molecular pathways of WSF on NAFLD in this study.

According to expert consensus, dietary disorders (i.e., overeating of fat and sweets), more leisure with less labor, emotional disorders, physical weakness from prolonged illness and insufficient endowment were considered as the primary causes of NAFLD (Zhang and Li 2017). Epidemiology statistics also revealed that excessive sugar and lipid intake, which could increase the metabolic burden of the liver, were major risk factors for NAFLD (Romero-Gómez et al. 2017). An increase in insulin resistance degree and hepatic fat storage is positively correlated with changes in plasma saturated fatty acids (Rosqvist et al. 2014). Moreover, excessive intake of sugar-sweetened beverages also has a positive correlation with fatty liver incidence and serum ALT levels (Ma et al. 2015). Therefore, the development of NAFLD models predominantly relies on dietary interventions with high-fat and high-sugar regimens to emulate the detrimental effects of an unhealthy human diet, except for drug-induced liver damage or unique strains of animals (Fang et al. 2022). In this study, we found that the HSHF diet would induce typical symptoms of NAFLD similar to those in humans like obesity, lipid deposition in organs, increased transaminase levels, lipid metabolism disorder, hepatocellular degeneration and hepatic inflammatory cell infiltration, as reported in other studies (Chen et al. 2017; Porras et al. 2017).

In this study, we revealed a noteworthy therapeutic impact of WSF across multiple dimensions on NAFLD treatment. Obesity ranks as a primary risk factor in the development of NAFLD. Meta-analysis results illustrated that 69.99% of individuals in the overweight category (BMI ≥ 25.0 kg/m^2^) exhibited NAFLD prevalence, and in the obese population (BMI ≥ 30.0 kg/m^2^), it increased further to 75.27% (Quek et al. 2023). We found that WSF decreased the body weight significantly of model mice from 7 weeks after administration compared to the MC group, and the weight gain of 17 weeks also decreased with significance. Additionally, liver and epididymis adipose mass weight declined significantly in the model mice, implying less lipid deposition in organs. Clinically, the disease progression of NAFLD is positively correlated with the serum levels of AST and ALT (Chinese Society of Endocrinology et al. 2018). It was identified that WSF had salient hepatoprotective effects due to reduced serum levels of AST and ALT in our study. Despite its inherent constraints, liver biopsy continues to be the gold standard for the course of NAFLD diagnosis and prognosis (Wang and Malhi 2018). H&E and Oil Red O staining results demonstrated that WSF could reverse pathological syndromes like steatosis, ballooning degeneration and inflammatory cell aggregation of hepatocytes caused by the HSHF diet. Meanwhile, NAS score reduction further proved the protective effects of WSF on hepatocyte lesions.

In 2020, an international panel advocated rechristening NAFLD as metabolic associated fatty liver disease (MAFLD) in an effort to underscore the metabolic underpinnings of the NAFLD pathogenesis course (Eslam et al. 2020). Our results showed that WSF could improve lipid metabolism by significantly modulating the serum and liver levels of TG and HDL-c, but it had no obvious effects on TC and LDL-c. Researches have indicated that excessive intake of bile salts not only causes an increase in intestinal absorption of cholesterol but also reduces the excretion of cholesterol by inhibiting the conversion to bile acids, leading to excessive accumulation of cholesterol in the plasma and liver of the organism (Song et al. 2015; Xiao et al. 2023; Tilg et al. 2022). LDL is the primary carrier of cholesterol in plasma, transporting cholesterol from the liver to peripheral tissues, and accumulation of cholesterol-enriched LDL is a hallmark of hypercholesterolemia (Islam et al. 2022). Moreover, clinical research data have also demonstrated that high hepatic and plasma cholesterol accompanied high levels of LDL-c with were seen in obeticholic acid administration patients (Neuschwander-Tetri et al. 2015). Our experimental observations revealed that neither the positive drug nor the WSF manifested substantial reductions in serum or liver TC levels and serum LDL-c levels. This lack of significant reduction implies that the cholesterol and sodium cholate-supplemented diet has elicited an extremely severe perturbation in the organism’s cholesterol metabolism. In conclusion, these results denoted that WSF could exert its anti-NAFLD efficacy mainly by ameliorating lipid metabolism partially and recovering liver inflammation and steatosis injury.

Furthermore, WSF exhibits a significant dose-dependent regulatory effect on glucose and lipid metabolism in rats with metabolic disorders, akin to the manifestations observed in NAFLD mice. However, the precise dose-dependent relationship of WSF in NAFLD models remains to be definitively established, due to variations in experimental animal strains and dietary compositions. Concurrently, WSF does not demonstrate significant toxic effects in normal mice at a dosage 12 times that of the human clinical dose. The observed significant differences in body weight may be attributed to the relatively higher initial body weight of the WS mice or potentially due to WSF enhancing their metabolic efficiency. The underlying mechanism warrants further investigation, particularly focusing on the activities of enzymes involved in glycolipid metabolism.

Network pharmacology has emerged as a potent strategy for holistically discerning the therapeutic targets of drugs in relation to diseases, owing to its capability of synthesizing colossal datasets to conduct virtual screenings grounded in both TCM ingredients and correlated symptoms (Zhong et al. 2023; Zhang et al. 2023). In the study combined with HPLC-Q-TOF/MS and network pharmacology, we attained 72 active components and 254 intersection target proteins. Sequentially, we further obtained NAFLD-related hub genes including TNF, IL6, AKT1, IL1B, PPARG and PTGS2, which played important roles in NAFLD abnormal metabolic pathways, corresponding to the degree values of the PPI interaction network. We have noticed that TNF-α, IL-6, IL-1β and PTGS2 (COX2) are all inflammatory factors, as well as inflammation is also supposed to be an important driver of NAFLD and the progression to NASH (Rohm et al. 2022; Luo and Lin 2021). Thus, we further gauged the gene counts and confidence levels of inflammation related pathways. The KEGG analysis showed that all the gene counts of TNF, IL − 17, Toll-like receptor and NF − kappa B signaling pathway were all greater than 15 with *P* values less than 0.01, indicating the key role of these pathways of WSF on NAFLD treatment. Therefore, inflammatory related TLR4 and NF − κB were also considered as the pivotal target proteins of WSF to alleviate NAFLD.

The outcomes from molecular docking analyses revealed that crucial components in WSF demonstrated high binding activities with pivotal targets comprised in inflammation related pathways (including TLR4, NF-κB, COX-2, IL-1β, IL-6 and TNF-α), with all minimum binding energies recorded at less than -5.0 kcal/mol (Tong et al. 2021; Zhou et al. 2022). This further confirmed the critical role of inflammatory pathways acquired from network pharmacology, particularly the TLR4/NF-κB/COX-2 pathway, in the molecular mechanism of WSF on NAFLD treatment. Furthermore, we found that the majority of active components with both lower binding energies to proteins and higher degree values in the network belonged to the saponins of *Gynostemmae Herba*. Our prior researches have similarly demonstrated that gypenosides could ameliorate hepatic inflammation in high-fat diet induced NAFLD rats by attenuating the LPS/TLR4/MyD88/NF-κB signaling pathway (Shen et al. 2020, 2022). Moreover, gypenosides could modulate associated enzyme functions involved in cholesterol production, fatty acid synthesis, transportation and degradation to improve lipid disorder in NAFLD as well (Zhou et al. 2023; Cheng et al. 2024). Those above implied that gypenosides could be the core bioactive ingredients of WSF, which need in-depth study later. In conclusion, WSF might exert pharmacological effects against NAFLD by mediating activities of inflammatory related proteins of TLR4/NF-κB/COX-2 pathway, which would be verified in the subsequent experiments.

The hepatic inflammation response is a crucial feature of NAFLD, and the canonical TLR4/NF-κB inflammatory pathway has been identified as a critical contributor in the advancement of NAFLD (Tang et al. 2023). In normative hepatocytic contexts devoid of stimulatory cues, NF-κB complexes with its cytoplasmic inactivator, the inhibitor of NF-κB (IκB), to form a suppressive ensemble that curtails the transcriptional activation potential of NF-κB (Wei et al. 2022). Upon stimulation of hepatocytes, TLR4 engages with the downstream adaptor protein of myeloid differentiation factor 88 (MyD88) and initiates a signaling cascade that subsequently activates the IκB kinase (IKK). This activation further prompts the phosphorylation and degradation of the IκB protein, leading to a dissociation of the NF-κB/IκB protein complex, which is crucial for the transcriptional regulation of inflammatory responses (Feng et al. 2020). Moreover, NF-κB activation leads to the production of pro-inflammatory cytokines comprising IL-1β, IL-6 and TNF-α, which are prominently implicated in the progression of NAFLD (Liu et al. 2019; Lv et al. 2023).

Experimental evidence has illustrated the impairment of linoleic and arachidonic acid metabolism in the liver of NAFLD mice induced by the high fat diet with a significant upregulation of hepatic TLR4 expression, leading to a substantial accumulation of inflammatory cytokines (Qin et al. 2023). Linoleic acid is categorized as an n-6 polyunsaturated fatty acids, whose downstream metabolite is arachidonic acid in vivo, and holds a significant role in mediating inflammatory processes (Burns et al. 2018). COX-2 is a formidable enzyme that facilitates the conversion of arachidonic acid into prostaglandins and instigates inflammation subsequent to activation by a myriad of inflammatory stimuli encompassing cytokines and bacteria (Hu et al. 2020). It has been manifested that excessive linoleic acid intake could result in liver steatosis, inflammation injury and fibrotic response with higher levels of hepatic TG and free fatty acids (FFAs) (Graham et al. 2023). Moreover, it would also promote NF-кB translocation and COX-2 activation, and induce production of proinflammatory cytokines like IL-1β, IL-6 and TNF-α by the release of arachidonic acid derived compounds (Marchix et al. 2015).

Our results revealed a marked decrease in the expression levels of TLR4, NF-κB and COX-2 in the liver tissue by WSF intervention compared with the NAFLD mice. As integrators of the inflammatory pathway in NAFLD, inactivation of NF-κB and COX-2 could lead to less secretion of pro-inflammatory cytokines like IL-1β, IL-6 and TNF-α (Huang et al. 2019; Cheng et al. 2013), which was consistent with the ELISA results in this study. Combined with the previous results of HPLC-Q-TOF/MS, bioinformatics and molecular docking, we supposed that WSF treatment could ameliorate hepatic lipid disorder and inflammation injury in NAFLD through regulation of the TLR4/NF-κB/COX-2 signaling pathway.

## Conclusions

This study indicated that WSF could be used to alleviate NAFLD caused by the HSHF diet via improving fat accumulation, lipid metabolism, liver function and pathological damage. Moreover, a new insight was provided into the mechanisms through the TLR4/NF-κB/COX-2 pathway combined with HPLC-Q-TOF/MS, bioinformatics, molecular docking and validation results. In addition, these results offer a pharmacological foundation for further product development and clinical implementation of WSF.

## Supplementary Information

Additional file 1.

Additional file 2.

## Data Availability

No datasets were generated or analysed during the current study.

## Abbreviations

ALT: Alanine aminotransferase
ASPL: Average shortest path length
AST: Aspartate transaminase
BNC: Betweenness centrality
BP: Biological process
CC: Cellular component
CNC: Closeness centrality
COX-2: Cyclooxygenase 2
DL: Drug-like properties
EA: Epididymis adipose
GLU: Glucose
GO: Gene ontology
HDL-c: High density lipoprotein cholesterol
HSHF: High-sucrose and high-fat
IHC: Immunohistochemistry
IL-1β: Interleukin-1β
IL-6: Interleukin-6
KEGG: Kyoto encyclopedia of genes and genomes
LDL-c: Low density lipoprotein cholesterol
MF: Molecular function
NAFLD: Nonalcoholic fatty liver disease
NAS: NAFLD activity score
NF-κB: Nuclear factor-kappa B
OB: Oral bioavailability
PPI: Protein–protein interaction
RT: Retention time
TC: Total cholesterol
TCM: Traditional Chinese medicine
TG: Triglyceride
TIC: Total ion chromatogram
TLR4: Toll-like receptor 4
TNF-α: Tumor necrosis factor α
WB: Western blot
WSF: Wang’s empirical formula
