# Supplementary material for: Bioinformatics based exploration of the anti-NAFLD mechanism of Wang’s empirical formula via TLR4/NF-κB/COX2 pathway
Source: Mol Med. 2024 Dec 27;30:278. doi: 10.1186/s10020-024-01022-3 (PMC11673956; doi:10.1186/s10020-024-01022-3)
Supplement: Supplementary file 1 — Additional file 1. [file 10020_2024_1022_MOESM1_ESM.docx]

**Supplementary Information**

**Bioinformatics based exploration of the anti-NAFLD mechanism of Wang’s empirical formula via TLR4/NF- κB/COX2 pathway**

Suhong Chen ^1, 3, 4^ ^†^, Chuanjie Zhou ^1, 3 †^, Jiahui Huang ^1, 3^ ^†^, Yunlong Qiao ^1,^ ^3 †^, Ning Wang ^1, 3^, Yuzhen Huang ^1,^ ^3^, Bo Li ^1,^ ^3^, Wanfeng Xu ^1, 3^, Xinglishang He ^1, 3^, Kungen Wang ^2, 5 *^, Yihui Zhi ^2, 5 *^, Guiyuan Lv ^4 *^ and Shuhua Shen ^2, 5 *^

^1^ Collaborative Innovation Center of Yangtze River Delta Region Green Pharmaceuticals, No. 18, Chaowang Road, Gongshu District, Zhejiang University of Technology, Hangzhou, Zhejiang 310014, China

^2^ The First Affiliated Hospital of Zhejiang Chinese Medical University, Hangzhou, Zhejiang, 310006, China

^3^ Zhejiang Provincial Key Laboratory of TCM for Innovative R & D and Digital Intelligent Manufacturing of TCM Great Health Products. Huzhou, Zhejiang 313200, China

^4^ College of Pharmaceutical Science, No. 548, Binwen Road, Binjiang District, Zhejiang Chinese Medical University, Hangzhou, Zhejiang 310053, China

^5^ Kun-Gen Wang National Famous Chinese Medicine Doctor Studio, Hangzhou, Zhejiang, 310006, China

† Suhong Chen, Chuanjie Zhou, Jiahui Huang and Yunlong Qiao shared co-first authorship.

^*^ Correspondence: ShuHua Shen: linda0358@163.com; GuiYuan Lv: zjtcmLgy@163.com; YiHui Zhi: medcat4@163.com; Kun-Gen Wang: wkg1220@ 163.com.

**Table.S1** The catalog numbers of the reagents

| **No.** | **Reagents** | **Catalog number** | **Manufacturer** |
| --- | --- | --- | --- |
| 1 | Essentiale (PPC) | BBJD209B | Sanofi - Aventis Pharmaceutical Co., Ltd. |
| 2 | TC kit | R202 | Ningbo Medical System Biotechnology Co., Ltd |
| 3 | TG kit | R201 |  |
| 4 | HDL-c kit | R203T |  |
| 5 | GLU kit | R108 |  |
| 6 | AST kit | R001 |  |
| 7 | ALT kit | R002 |  |
| 8 | Hematoxylin-Eosin reagent | R20570-2 | Shanghai Yuanye Biotechnology Co., Ltd. |
| 9 | Oil red O reagent | A600395 | BBI Life Science Corporation |
| 10 | TC assay kit for liver | A111-1-1 | Nanjing Jiancheng Bioengineering Institute |
| 11 | TG assay kit for liver | A110-1-1 |  |
| 12 | ELISA kit of IL-1β | MM-0040M1 | Jiangsu Meimian Industrial Co., Ltd. |
| 13 | ELISA kit of TNF-α | MM-0132M1 |  |
| 14 | Instant immunohistochemistry kit | SA1020 | Wuhan Boster Biological Technology Co., Ltd. |
| 15 | DAB staining kit | P0203 | Beyotime Biotechnology Reagent Co., Ltd. |
| 16 | BCA assay kit | P0010 |  |
| 17 | RIPA buffer | P0013B |  |
| 18 | Protein free rapid blocking buffer | P0252 |  |
| 19 | COX2 antibody | 12375-1-AP | Proteintech Group Inc. |
| 20 | IL-6 antibody | 66146-1-Ig |  |
| 21 | Secondary antibodies | RGAR001/  RGAM001 |  |
| 22 | TLR4 antibody | bs-20594R | Beijing Biosynthesis Biotechnology Co., Ltd. |
| 23 | NF-κB antibody | T55034F | Hangzhou DiagBio Biotechnology Co., Ltd |
| 24 | β-actin antibody | GB15001-100 | Wuhan Servicebio Technology Co., Ltd. |
| 25 | Enhanced chemiluminescent assay kit | TE0015 | Nature Biosciences Ltd. |

Animal experiment procedure of WSF administration on rats with glucose and lipid metabolic disorders

SD rats (male, n=20) were purchased from Shanghai Slac Laboratory Animals Co., Ltd. (SCXK(Hu)2022-0004, Shanghai, China). All the animals were reared in standardized environmental conditions characterized by a 12-hour light-dark photoperiod with unrestricted access to water and food. The animal procedures were meticulously conducted in strict adherence to the Zhejiang University of Technology’s Guidelines for the Care and Use of Laboratory Animals.

After adaptive feeding for 3 days, a total of twenty SD rats were randomly assigned into five groups according to their body weight with the random number method: the normal group (NC), the model group (MC), WSF low-dose administration group (WS-L, 3.56 g/kg), WSF median-dose administration group (WS-L, 7.13 g/kg), and WSF high-dose administration group (WS-H, 14.26 g/kg), each consisting of four rats. The NC rats was fed with standard diet, while rats in the MC and WS were both received a high-fructose and high-fat (HFHF) diet over a period of 18 weeks. The WS rats were daily administered WSF at various dose (i.g.) adjusted to the volume of 1 ml/100 g in accordance with the body weight after modeling for 10 weeks. The HFHF diet (TP0860), consisting of 15% fat and 15% fructose was derived from Nantong Trophic Feed Technology Co., Ltd (Nantong, China).

Upon conclusion of the experiment, mice were subjected to an overnight fasting before blood was drawn from the ocular venous plexus. Obtained blood samples were centrifuged for 10 min twice at 3000 rpm to attain serum for biochemical analysis.

**Table.S2 Body weight change of** **rats with glucose and lipid metabolic disorders (g)**

| **Group** | **Week 0** | **Week 2** | **Week 4** | **Week 6** | **Week 8** |
| --- | --- | --- | --- | --- | --- |
| NC | 395.45±13.85** | 393.33±23.06** | 431.73±25.37** | 428.43±21.83** | 447.98±20.68** |
| MC | 537.73±34.14 | 532.53±32.85 | 545.80±39.51 | 541.88±38.11 | 545.15±33.47 |
| WS-L | 527.70±33.96 | 509.78±33.31 | 529.08±31.06 | 519.33±20.10 | 516.88±22.88 |
| WS-M | 512.60±28.90 | 502.73±26.58 | 513.43±31.58 | 505.60±37.18 | 504.35±36.50 |
| WS-H | 514.60±36.00 | 499.65±57.56 | 489.30±28.57* | 482.28±43.32* | 488.75±39.40* |

**Note:** **NC:** Normal control group; **MC:** Normal control group; **WS**-**L:** Low dosage (3.56 g/kg); **WS**-**M**: Mid dosage (7.13 g/kg); **WS-H**: High dosage (14.26 g/kg). All of values were presented as mean ± SD with significance markers of ^*^*P* < 0.05 and ^**^*P* < 0.01 vs MC group. (n=4)


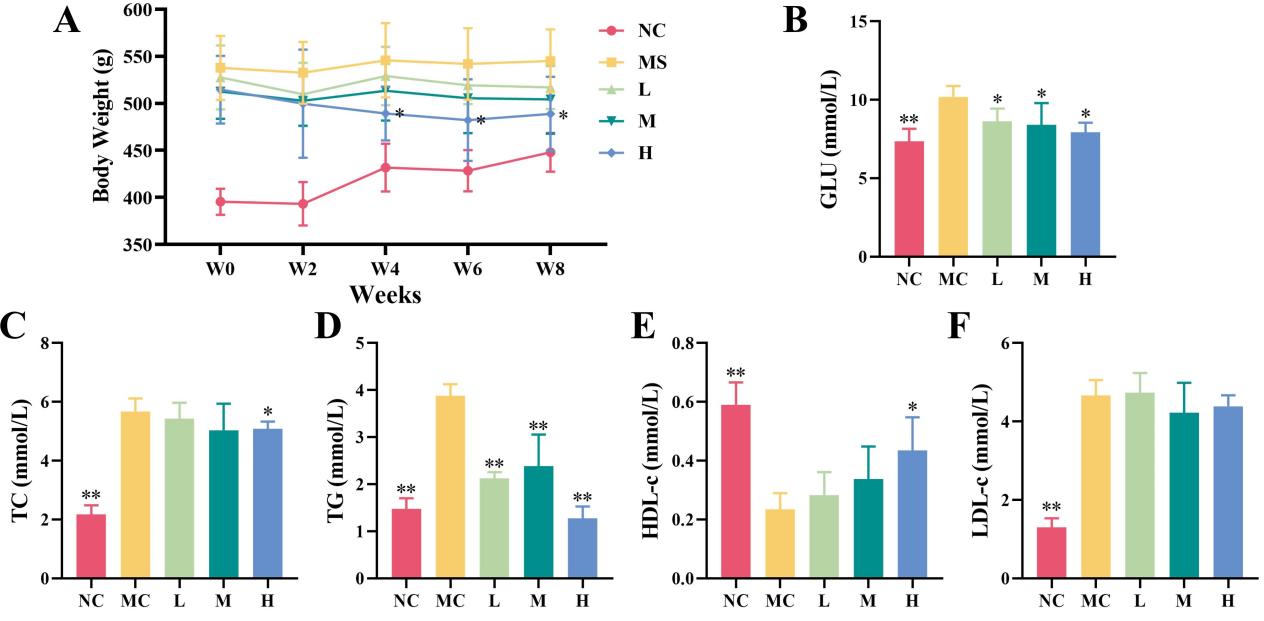


**Fig.S1 Body weight and serum biochemistry change of rats with glucose and lipid metabolic disorders**. (**A**) Body weight. (**B**) GLU level. (**C**) TC level. (**D**) TG level. (**E**) HDL-c level. (**F**) LDL-c level. **NC:** Normal control group; **MC:** Normal control group; **L:** Low dosage (3.56 g/kg) of WSF; **M**: Mid dosage (7.13 g/kg) of WSF; **H**: High dosage (14.26 g/kg) of WSF. All of values were presented as mean ± SD with significance markers of ^*^*P* < 0.05 and ^**^*P* < 0.01 vs NC group. (n=4)

Conclusion of WSF administration on rats with glucose and lipid metabolic disorders

After 10 weeks of HFHD feeding to establish the model, the body weight and serum levels of GLU, TC, TG, and LDL-c in the model rats were significantly increased (*P* < 0.05, Student's t-test, the same below), while the level of HDL-c was significantly decreased (*P* < 0.05), indicating that the model rats exhibited disorders in glucose and lipid metabolism. After 8 weeks of administration with WSF at a dose of 14.26 g/kg, the body weight and serum levels of GLU, TC, and TG in the model rats were significantly reduced (*P* < 0.05), while the HDL-c level was increased (*P* < 0.05). In contrast, administration of WSF at doses of 3.56 and 7.13 g/kg was only effective in modulating serum GLU and HDL-c levels. More importantly, with the continuous increase in the dosage of administration, the improvement effects on body weight and serum levels of GLU, TC, and HDL-c gradually enhanced, exhibiting a certain dose-dependency.

In conclusion, WSF demonstrates a notable dose-dependent regulatory effect on glucose and lipid metabolism in rats with metabolic disorders, with **the optimal dosage identified as 14.26 g/kg for rats.**

Animal experiment procedure of 28-day oral toxicity experiment

ICR mice (male, n=20) were purchased from Hangzhou Qizhen Experimental Animal Technology Co., Ltd. (SCXK(Zhe)2022-0005, Hangzhou, China). All the animals were reared in standardized environmental conditions characterized by a 12-hour light-dark photoperiod with unrestricted access to water and food. The animal procedures were meticulously conducted in strict adherence to the Zhejiang University of Technology’s Guidelines for the Care and Use of Laboratory Animals.

After adaptive feeding for 1 week, a total of twenty ICR mice were randomly assigned into two groups according to their body weight with the random number method: the normal group (NC), WSF administration group (WS, 28.53 g/kg), each consisting of ten mice. The NC and WS mice were both fed with standard diet over a period of 4 weeks. The WS mice were daily administered WSF at a dose of 28.53 g/kg (i.g., 12 times of clinical dose) adjusted to the volume of 1 ml/100 g in accordance with the body weight for whole 4 weeks.

Upon conclusion of the experiment, mice were subjected to an overnight fasting before blood was drawn from the ocular venous plexus. Obtained blood samples were centrifuged for 10 min twice at 3000 rpm to attain serum for biochemical analysis. The mice were subsequently anesthetized (isoflurane inhalation) with removing their liver as quickly as possible.


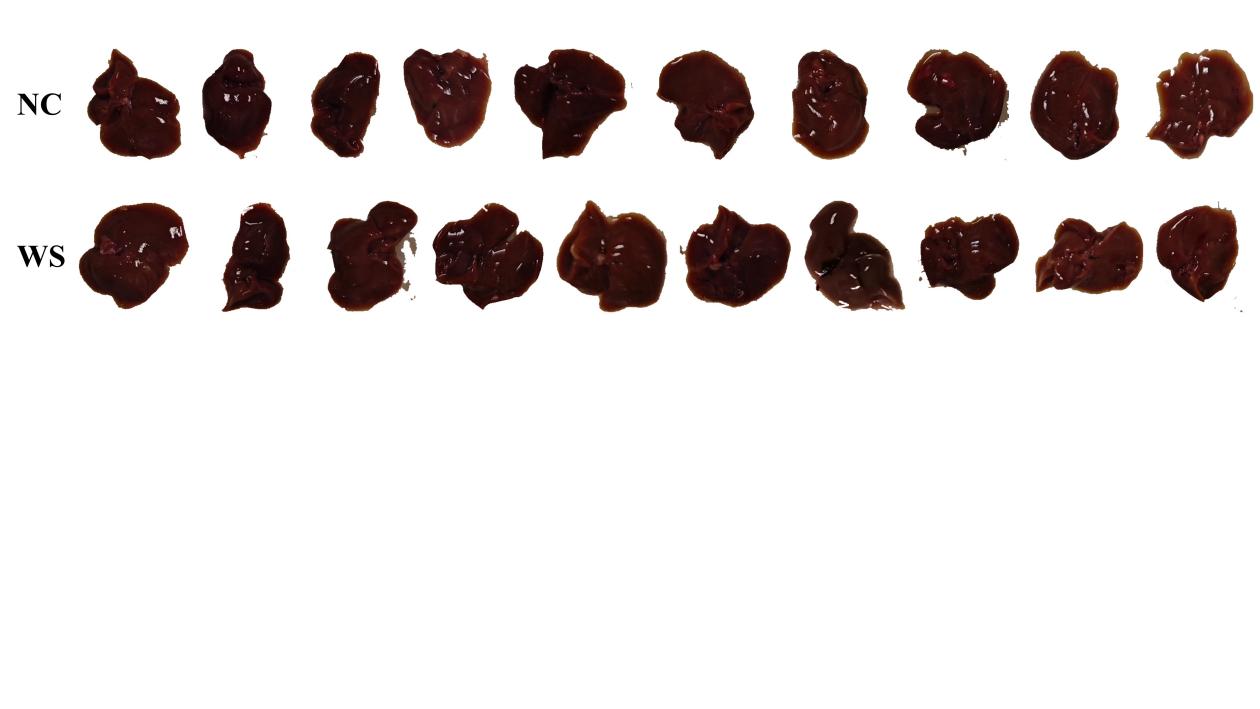


**Fig.S2** General view of mice livers from 28-day oral toxicity experiment of WSF. **NC:** Normal control group; **WS:** WSF administration group (28.53 g/kg). (n=10)


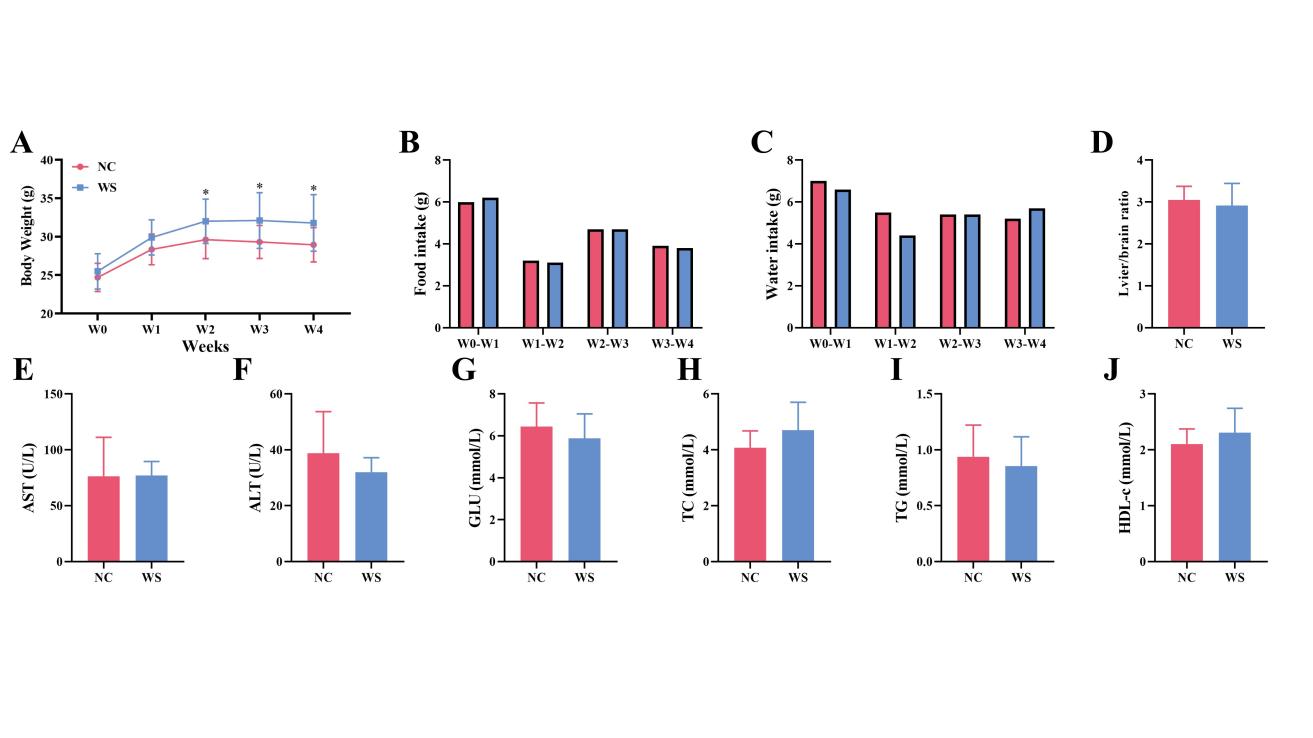


**Fig.S3** Results of 28-day oral toxicity experiment of WSF. (**A**) Body weight. (**B**) Food intake. (**C**) Water intake. (**D**) Liver brain ration. (**E**) AST level. (**F**) ALT level. (**G**) GLU level. (**H**) TC level. (**I**) TG level. (**J**) HDL-c level. Most of values were presented as mean ± SD with significance markers of ^*^*P* < 0.05 and ^**^*P* < 0.01 vs NC group. (n=10)

Conclusion of 28-day oral toxicity experiment

After 28 days of administration of WSF at 12 times the clinical dose for humans, we found that there was no significant difference in food intake, water intake, liver/brain ratio, and serum levels of AST, ALT, GLU, TC, TG, and HDL-c in all mice. Additionally, the body weight of WS mice was significantly higher than that of NC mice, starting from the second week (*P* < 0.05, Student’s t-test). It is speculated that this may be related to the relatively high initial body weight of the WS mice or that WSF improves their metabolic efficiency. The specific mechanism still requires further study on the activities of enzymes related to glycolipid metabolism.

In conclusion, **WSF has no significant toxic effect on normal mice at 12 times the clinical dose for humans**.

**Table.S3** Tissue dehydration procedure

| **No.** | **Procedure** | **Time** | **No.** | **Procedure** | **Time** |
| --- | --- | --- | --- | --- | --- |
| 1 | 50% Ethanol | 60 min | 7 | Anhydrous ethanol II | 30 min |
| 2 | 75% Ethanol | 60 min | 8 | Anhydrous ethanol : Dimethylbenzene = 1 : 1 | 15 min |
| 3 | 85% Ethanol | 60 min | 9 | Dimethylbenzene I | 10 min |
| 4 | 95% Ethanol I | 30 min | 10 | Dimethylbenzene II | 10 min |
| 5 | 95% Ethanol II | 30 min | 11 | Paraffin wax I | 60 min |
| 6 | Anhydrous ethanol I | 30 min | 12 | Paraffin wax II | 60 min |

**Table.S4** Tissue H&E stain procedure

| **No.** | **Procedure** | **Time** | **No.** | **Procedure** | **Time** |
| --- | --- | --- | --- | --- | --- |
| 1 | Xylene dewaxing I | 10 min | 13 | Tap water wash | 1 min |
| 2 | Xylene dewaxing II | 10 min | 14 | Turn blue with 1% dilute ammonia water | 30 s |
| 3 | Anhydrous ethanol I | 2 min | 15 | Tap water wash | 1 min |
| 4 | Anhydrous ethanol II | 2 min | 16 | Eosin staining | 1 min |
| 5 | 95% Ethanol I | 2 min | 17 | Tap water wash | 30 s |
| 6 | 95% Ethanol II | 2 min | 18 | 80% Ethanol | 20 s |
| 7 | 70% Ethanol | 2 min | 19 | 80% Ethanol | 30 s |
| 8 | 50% Ethanol | 2 min | 20 | Anhydrous ethanol I | 2 min |
| 9 | Tap water wash | 2 min | 21 | Anhydrous ethanol II | 2 min |
| 10 | Hematoxylin staining | 6 min | 22 | Transparency with xylene I | 10 min |
| 11 | Tap water wash | 1 min | 23 | Transparency with xylene II | 10 min |
| 12 | 1% Hydrochloric acid in 75% ethanol differentiation | 10 s |  |  |  |

**Table.S5** The catalog and lot numbers of the antibodies

| **No.** | **Antibody** | **Catalog number** | **Lot number** |
| --- | --- | --- | --- |
| 1 | TLR4 | bs-20594R | BC03078334 |
| 2 | NF-κB | T55034F | 10144146 |
| 3 | COX2 | 12375-1-AP | 00072808 |
| 4 | IL-6 | 66146-1-Ig | 10017944 |

Code for Image J v1.54f:

1. **Code for statistical analysis of liver parenchymal cell area:**

run("8-bit");

setAutoThreshold("Default");

//run("Threshold...");

setThreshold(35, 162, "raw");

//setThreshold(35, 162);

setOption("BlackBackground", false);

run("Convert to Mask");

run("Measure");

1. **Code for statistical analysis of Oil Red O area:**

title=getTitle()

run("Colour Deconvolution", "vectors=RGB");

run("8-bit");

selectImage(title+ "-(Colour_1)");

setAutoThreshold("Default no-reset");

//run("Threshold...");

//setThreshold(0, 47);

run("Convert to Mask");

run("Measure");

**Oil Red O area ratio (%) = Oil Red O area / Liver parenchymal cell area × 100%**
